# Wood–Ljungdahl pathway encoding anaerobes facilitate low-cost primary production in hypersaline sediments at Great Salt Lake, Utah

**DOI:** 10.1093/femsec/fiae105

**Published:** 2024-07-25

**Authors:** Anna Shoemaker, Andrew Maritan, Su Cosar, Sylvia Nupp, Ana Menchaca, Thomas Jackson, Aria Dang, Bonnie K Baxter, Daniel R Colman, Eric C Dunham, Eric S Boyd

**Affiliations:** Department of Earth Sciences, Montana State University, P.O. Box 173480, Bozeman, MT 59717, United States; Department of Microbiology and Cell Biology, Montana State University, P.O. Box 173520, Bozeman, MT 59717, United States; Department of Microbiology and Cell Biology, Montana State University, P.O. Box 173520, Bozeman, MT 59717, United States; Department of Chemistry and Biochemistry, Montana State University, P.O. Box 173400, Bozeman, MT 59717, United States; Department of Microbiology and Cell Biology, Montana State University, P.O. Box 173520, Bozeman, MT 59717, United States; Department of Microbiology and Cell Biology, Montana State University, P.O. Box 173520, Bozeman, MT 59717, United States; Department of Chemistry and Biochemistry, Montana State University, P.O. Box 173400, Bozeman, MT 59717, United States; Great Salt Lake Institute, Westminster University, 1840 South 1300 East, Salt Lake City, UT 84105, United States; Department of Microbiology and Cell Biology, Montana State University, P.O. Box 173520, Bozeman, MT 59717, United States; Department of Microbiology and Cell Biology, Montana State University, P.O. Box 173520, Bozeman, MT 59717, United States; Department of Microbiology and Cell Biology, Montana State University, P.O. Box 173520, Bozeman, MT 59717, United States

**Keywords:** acetogen, acetothermia, *Ca. Bipolaricaulia*, carbon fixation, primary production, thermoplasmatota

## Abstract

Little is known of primary production in dark hypersaline ecosystems despite the prevalence of such environments on Earth today and throughout its geologic history. Here, we generated and analyzed metagenome-assembled genomes (MAGs) organized as operational taxonomic units (OTUs) from three depth intervals along a 30-cm sediment core from the north arm of Great Salt Lake, Utah. The sediments and associated porewaters were saturated with NaCl, exhibited redox gradients with depth, and harbored nitrogen-depleted organic carbon. Metabolic predictions of MAGs representing 36 total OTUs recovered from the core indicated that communities transitioned from aerobic and heterotrophic at the surface to anaerobic and autotrophic at depth. Dark CO_2_ fixation was detected in sediments and the primary mode of autotrophy was predicted to be via the Wood–Ljungdahl pathway. This included novel hydrogenotrophic acetogens affiliated with the bacterial class *Candidatus Bipolaricaulia*. Minor populations were dependent on the Calvin cycle and the reverse tricarboxylic acid cycle, including in a novel *Thermoplasmatota* MAG. These results are interpreted to reflect the favorability of and selectability for populations that operate the lowest energy requiring CO_2_-fixation pathway known, the Wood–Ljungdahl pathway, in anoxic and hypersaline conditions that together impart a higher energy demand on cells.

## Teaser

Anaerobes that facilitate primary production in polyextremophilic hypersaline sediments encode the lowest cost autotrophic pathway, the Wood–Ljungdhal pathway, to minimize energy expenditures.

## Introduction

Autotrophs form the base of aquatic food webs and have a central role in energy flow to secondary consumers (Lindeman 1942). Thus, autotrophs and their activities influence the overall productivity of aquatic ecosystems and their taxonomic and functional biodiversity (Smith 2007). In the photic zone of hypersaline lakes, the dominant primary producers are microbial, and include oxygenic photosynthetic organisms including both algae such as *Dunaliella* (Oren 2005) and *Cyanobacteriota* such as *Euhalothece* (Brock 1976, Kanik et al. 2020) as well as anoxygenic photosynthetic bacteria such a members of the *Chromatiaceae* (Imhoff 2001). Far less is known of the primary producers in aphotic and benthic regions of hypersaline environments. Given the lower solubility of oxygen (O_2_) in hypersaline waters (Garcia and Gordon 1992), aphotic and benthic regions of hypersaline environments are likely to be suboxic to anoxic. Together, with hypersalinity and associated low water activity (Grant 2004), low O_2_ conditions would lead to polyextremophilic conditions (Capece et al. 2013, Merino et al. 2019) that could increase energy demands on cells (Hoehler 2007, Shock and Holland 2007).

Hypersaline aquatic environments are typically formed through evaporative processes (Grant 2004), and depending on their geological setting can vary widely in their ionic composition and in the availability of electron donors and acceptors (Oren 2013). Among the most widely available electron acceptors in anoxic zones of hypersaline aquatic environments are sulfate (SO_4_^2−^) and dissolved inorganic carbon [DIC; Σ dissolved carbon dioxide (CO), carbonic acid (H_2_CO_3_), bicarbonate (HCO_3_^−^), and carbonate (CO_3_^2−^), respectively]. The upper salinity limit for a variety of dissimilatory microbial processes, including those dependent on SO_4_^2−^ (i.e. SO_4_^2−^reducers) and DIC (i.e. acetogens and methanogens) have been compiled based on laboratory studies of cultivars or via measurements of microbial activities associated with natural samples (Oren 1999, 2013). The salinity limit for dissimilatory SO_4_^2−^ reducers varies depending on whether they are complete or incomplete organic carbon oxidizers. Complete organic carbon oxidizers, or those that oxidize organic substrates (e.g. acetate) completely to CO_2_, are apparently restricted to <12% salt (Brandt and Ingvorsen 1997). In contrast, incomplete oxidizers, or those that only partially oxidize organic carbon substrates, have been identified at salinities up to 30% salt (Brandt et al. 2001), suggesting incomplete oxidizers can outcompete the complete oxidizers in high salt conditions. Autotrophic acetogens and methanogens that generate acetate and methane from H_2_ and CO_2_, respectively, have upper salt tolerances of 24% and 11% (Zhilina et al. 1996, Ollivier et al. 1998), respectively. This suggests the possibility that acetogens can outcompete methanogens at elevated salt. These differences have been attributed to differences in the energy yields of those respective metabolisms and competition for the same electron donors/acceptors, namely H_2_ and CO_2_ (Oren 2013). As such, methanogens are thought to be more dependent on methylated compounds in hypersaline systems to minimize competition with acetogens for H_2_/CO_2_ and to enable their coexistence (Kato et al. 2014).

Recent cultivation-independent studies have identified putative SO_4_^2−^ reducers in the north arm of GSL, Utah (Dunham et al. 2020) and the nearby shallow subsurface halite-saturated sediments of the Bonneville Salt Flats (BSL), Utah (McGonigle et al. 2022a). These organisms were closely related to the chemolithotrophic and hydrogenotrophic SO_4_^2−^ reducing genera *Desulfovermiculus* (Beliakova et al. 2006) and *Desulfonauticus* (Mayilraj et al. 2009). Further, acetogens that were classified to the class *Candidatus Bipolaricaulia* (formerly referred to as candidate division Acetothermia or candidate division OP1; Hugenholtz et al. 1998, Hao et al. 2018) were identified in the BSL and GSL sediment columns. The abundance of 16S rRNA gene transcripts affiliated with *Ca. Bipolaricaulia* was shown to increase with depth in the GSL sediment column (Dunham et al. 2020) and the relative abundance of reads that mapped to a metagenome-assembled genome (MAG) affiliated with *Ca. Bipolaricaulia* was shown to increase with depth in the BSL sediments (McGonigle et al. 2022b). Here, we apply metagenomic sequencing to three depth intervals along a 30-cm sediment core from the north arm of GSL to determine the composition, structure, and function of communities in anoxic hypersaline (roughly ∼30% NaCl) sediments. Specifically, we seek to identify adaptations and putative trophic level interactions that enable habitation of these polyextreme environments.

## Methods

### Sample site description

**Figure 1.**
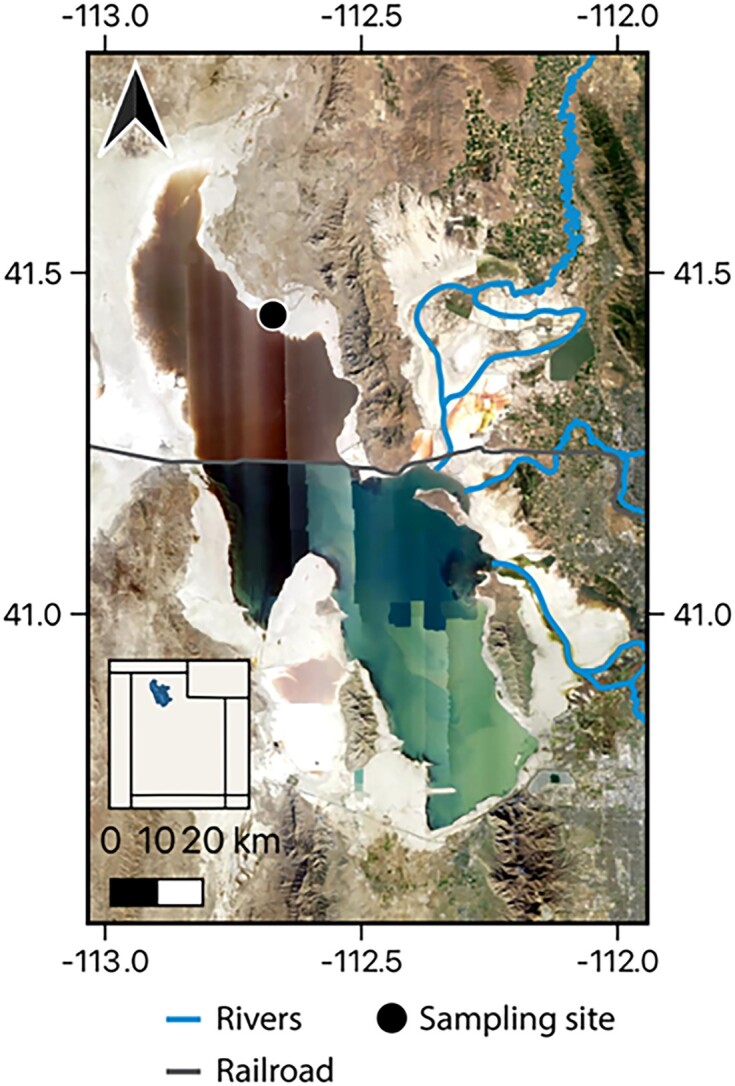
Map of GSL with the location in the NA, where the sediment column was collected indicated by a black dot. The railroad causeway separating the NA from the SA and the primary rivers draining into GSL are indicated. Base map National Agriculture Imagery Program (NAIP) imagery sourced from United States Department of Agriculture (USDA) and river shapefile modified from the National Hydrologic Dataset (NHD). State shapefile within the inlay map is from the United States Census Bureau’s Master Address File/Topologically Integrated Geographic Encoding and Referencing (MAF/TIGER) Database (MTDB).

GSL is a terminal lake in north-central Utah, USA (Hassibe and Keck 1991). It exhibits a range of salinities due to localized freshwater input (∼95% of the total) to the southern end of the lake (Belovsky et al. 2011) (Fig. 1). The salinity of GSL was 20%–27% between 1900 and 1959 (Stephens 1990). However, in 1959, a rock and gravel railroad causeway was constructed across the lake, severing the lake into what is often referred to as a north arm (NA) and south arm (SA) (Cannon and Cannon 2002) (Fig. 1). The flow of water between the two arms is restricted to a recently constructed breach (Brown et al. 2023), which results in increased salinities in the NA (>30%) and decreased salinities in the SA (typically <15%) (Baxter et al. 2005).

### Sample collection and geochemical analyses

The sediment core from the NA of GSL was collected, processed, and analyzed as part of a prior study (Dunham et al. 2020). Briefly, this sediment core was collected in Rozel Bay (41.43783°, −112.67103°) on 9 May 2016 (Fig. 1). Two cores were taken ∼5 m from the shoreline and where the sediment water interface was ∼10 cm below the water air interface. Briefly, cores were taken by driving polyvinyl chloride pipes (6.3 cm diameter) into the sediment to a depth of 50 cm. One core was frozen upright with dry ice on site and remained frozen during the transport to the lab, where it was transferred to a −80°C freezer. This core was sectioned into 5 cm segments (pucks) in a −20°C facility using a sterilized band saw. Core pucks were halved, with one half reserved for molecular (RNA) microbiological analysis and stored at −80°C, while the other half was used for geochemical measurements, as described previously (Dunham et al. 2020). A second core was collected and was stored upright on ice during transport back to the lab. This core was used for activity measurements. The sediment core was characterized extensively, with its minerology, porewater chemistry, and select microbial activities measured and reported previously (Dunham et al. 2020).

Previously measured rates of ^14^C-bicarbonate assimilation, porewater dissolved CO_2_ concentrations, and porewater pH values in a parallel sediment core from the NA of GSL (Dunham et al. 2020) were used to normalize rates of ^14^C-bicarbonate assimilation to rates of total DIC assimilation. Specifically, it was assumed that dissolved CO_2_ (measured) and bicarbonate (not measured) were in equilibrium in the sediment core (pKa = 6.3). The Henderson–Hasselbach equation, along with the measured pH, were then used to calculate the concentration of bicarbonate. Concentrations of dissolved CO_2_ and bicarbonate were then summed to estimate total DIC, which was then used to normalize rates of ^14^C-bicarbonate to rates of total DIC fixed. Rates of ^14^C-bicarbonate measured in disintegrations per minute (DPM) per gram dry-weight sediment (gdws) per hour were converted to µmols gdws^−1^ h^−1^ using the specific activity of the ^14^C bicarbonate (52 µCi µmol^−1^). This was multiplied by the ratio of the amount of ^14^C-labeled DIC added (1 µmol) to total DIC (from calculations above) to arrive at rates of total DIC assimilation.

### DNA extraction and metagenomic sequencing, assembly, and analyses

Sediment core pucks stored at −80°C were thawed at room temperature in an ethanol- and UV-treated laminar flow hood. Based on variation in community 16S rRNA gene transcript sequencing, in particular the relative abundance of transcripts associated with potentially novel *Ca. Bipolaricaulia* (Dunham et al. 2020), ∼1 g subsections of sediment pucks from depths of 0, 5, and 30 cm were subjected to DNA extraction using the FastDNA Spin Kit for Soil (MP Biomedicals, Irvine, CA) following the manufacturer’s instructions. Genomic DNA was quantified fluorometrically (170–520 ng per sample) via the high sensitivity Qubit assay (Thermo Fisher Scientific, Waltham, MA). Shotgun metagenomic sequencing was conducted on genomic DNA from sediment core samples. Illumina library preparation and paired-end sequencing (2 × 150 bp) were conducted at the Josephine Bay Paul Center, located at Marine Biological Laboratory at Woods Hole Marine Biological Laboratory in Woods Hole, Massachusetts using the Illumina NextSeq platform.

Reads were trimmed and down-sampled with the TrimGalore v.0.6.0 and BBMap programs to cleave sequencing adapters and remove sequencing redundancies as previously described (Payne et al. 2019). Trimmed and down-sampled sequences were assembled individually and coassembled using Spades v.3.14.0 specifying default parameters. The quality of the assemblies was then compared using various assembly metrics and the metaquast program (v.4.3) (Mikheenko et al. 2018). Assembly statistics are reported in Supplementary Table S1. The coassembled metagenomes resulted in substantially higher quality metagenome assemblies, and these were thus further used to characterize the communities. Assembly statistics are reported in Supplementary Table S1. Assembled contigs were binned into MAGs using MetaBAT v.0.26.3 (Kang et al. 2015) based on read depth and tetranucleotide frequency specifying the “verysensitive” setting. The assemblies were binned separately after coassembly and the quality, completeness, and level of contamination of each bin was assessed using CheckM v.1.0.5 (Parks et al. 2015). “Outlier” contigs were removed with RefineM v.0.0.23 (Parks et al. 2017). Only MAGs that exhibited >50% estimated completeness and <10% contamination (consistent with moderate to high quality genomes (Bowers et al. 2017)) were retained for further analyses. The MAGs were taxonomically classified using the GTDB-Tk v.1.3.0 (Chaumeil et al. 2019) classifier and the bac120 and arc122 datasets for bacterial and archaeal classification, respectively. Taxonomic designations are provided to the lowest taxonomic rank that was formally recognized at the time of the writing of this manuscript. The relative abundances of MAGs were estimated based on mapping of quality-filtered reads to those MAGs. Relative abundances are reported as the % of reads mapped to MAGs. MAG contigs have been deposited in the National Center for Biotechnological Information (NCBI) Whole Genome Sequence database under bioproject accession PRJNA1036658. MAG taxonomy, estimated completeness, contamination, and abundance are reported in Supplementary Tables S2 and S3.

MAGs from each depth were collapsed into operational taxonomic unit (OTUs) using a threshold of >95% average nucleotide identity (ANI), using the fastANI program (v.1.32) (Jain et al. 2018). A representative MAG from each OTU was selected for downstream analyses based on a set of empirically defined hierarchical criteria. MAGs were selected firstly to maximize estimated genome completion, secondly to minimize estimated contamination, and, when necessary, thirdly to maximize their relative abundance. MAG gene predictions were made using PROKKA v 1.11 (Seemann 2014) and resultant protein annotated files were then uploaded to the Kyoto Encyclopedia of Genes and Genomes (KEGG) database (Kanehisa and Goto 2000) using the KEGG Automatic Annotation Server (Moriya et al. 2007) to further examine the potential functionalities encoded by MAGs.

### Modes of carbon metabolism

MAGs were examined for key functionalities related to carbon metabolism using KEGG outputs and these were further verified manually with BLASTp using protein queries from organisms with those demonstrated capabilities (see Supplementary Tables S4 and S5). MAGs were first screened for pathways that allow for autotrophy. The six major autotrophic pathways are (1) the Calvin–Benson–Bassham (CBB) cycle, (2) the reductive tricarboxylic acid (rTCA) cycle, (3) the WL pathway, (4) the 3-hydroxypropionate (3HP) bicycle, (5) the 3-hydroxypropionate/4-hydroxybutyrate (3HP/4HB) cycle, and (6) the dicarboxylate/4-hydroxybutyrate (DC/4-HB) cycle (Berg et al. 2010, Berg 2011). Diagnostic proteins for each of these pathways were used to designate MAGs as being autotrophic. These included homologs of ribulose 1,5-bisphosphate carboxylase/oxygenase (RuBisCO; Enzyme Category (EC) 4.1.1.39) and phosphoribulokinase (PRK; EC 2.7. 1.19) for the CBB cycle and citryl-CoA synthetase (ccs; EC 6.2.1.18), citryl-CoA lyase (cit; EC 4.1.3.34), and ATP-citrate lyase (ACLY; EC 2.3.3.8) for the rTCA cycle. Homologs of carbon monoxide dehydrogenase/acetyl-CoA synthase (CODH/ACS; EC 1.2.7.4) and formate-tetrahydrofolate ligase (FHS; 6.3.4.3) were used to identify evidence for the WL pathway while homologs of propionyl-CoA carboxylase (PCC; EC 6.4.1.3), malonyl-CoA reductase (MCR; EC 1.2.1.75), malyl-CoA/B-methylmalyl-CoA/citranyl-lyase (MCL; EC 4.1.3.24), and acetyl-CoA carboxylase (ACC; 6.4.1.2) were used to identify evidence for the 3HP pathway. Evidence for the 3HP/4HB pathway was gained by identifying homologs of the same enzymes as the 3HP pathway with the addition of 4-hydroxybutyrl-CoA dehydratase (4-BUDH; EC 4.2.1.120). No enzyme homolog is diagnostic for the DC/4-HB pathway, since it uses enzymes common to the rTCA or 3HP/4B cycles above. However, the DC/4-HB pathway can be differentiated from the 3HP/4HB pathway by identifying homologs of pyruvate synthase (POR; EC 1.2. 7.1) (Havig et al. 2011, St Clair et al. 2019).

MAGs lacking homologs of these indicator proteins for autotrophic pathways were assigned as putatively heterotrophic. The carbon metabolism of cells was further assessed by examining MAGs for evidence of glycolytic, gluconeogenic, tricarboxylic acid, and pentose phosphate pathways, based on KEGG outputs. Putative autotrophic MAGs that also encoded glycolytic, TCA, and pentose phosphate pathways were defined as facultative autotrophs. Putative heterotrophs that lacked terminal oxidases (described below) but that had components of glycolytic pathways and fermentative capabilities (e.g. homologs of putative H_2_ evolving [NiFe]- or [FeFe]-hydrogenases) were classified as fermenters. While the approaches used to classify an organism as autotrophic or heterotrophic were conservative and used multiple lines of evidence where possible, it is important to note that these MAGs are incomplete and it is possible that the lack of a given protein homolog(s) in a genome is attributable to this.

### Usage of electron donors and acceptors

MAGs were screened for homologs of proteins allowing for use of select electron donors and acceptors based on KEGG outputs and BLASTp analyses. This included homologs of proteins involved in reversible H_2_ metabolism [NiFe]- and [FeFe]-hydrogenases, EC 1.12.1.2 or 1.12.99.6), arsenite oxidation (AioA, EC:1.20.9.1), sulfide oxidation (Sqo, EC 1.8.5.8), thiosulfate/sulfur oxidation (Sox, EC 2.8.5.2), and methane oxidation (MmoX, EC 1.14.13.25). Putative ([NiFe]- and [FeFe]-hydrogenases detected via BLAST search were manually assessed for the presence of distinguishing cysteine residues (Peters et al. 2015) and were classified using the HydDB tool (Søndergaard et al. 2016), with manual verification of ligand motifs (Peters et al. 2015). A complete list of key enzymes and their EC numbers that were used to screen MAGs is provided in Supplementary Table S6.

KEGG outputs and BLASTp analyses were used to identify homologs of proteins that would allow cells to incorporate O_2_ into their metabolism. This was principally assessed based on MAGs encoding homologs of cytochrome *c* oxidase (Cox I and II; EC 7.1.1.9). MAGs that did not encode Cox homologs were also screened for homologs of the cytochrome *bd* complex (CydABX; EC 7.1.1.7), since CydABX serves to reduce O_2_ for detoxification (currently only Bacteria). MAGs that encoded neither Cox nor the Cyd were deemed anaerobes and this was cross-checked (when possible) by examining other MAGs that correspond to a given OTU. Further, MAGs were screened for enzyme homologs that may allow use of alternative electron acceptors, including proteins involved in dissimilatory nitrate reduction (NarABG, EC 1.7.5.1 and NapAB, EC 1.9.6.1), sulfate/sulfite reduction (Sat, EC 2.7.7.4; AprAB, EC 1.8.99.2; DsrAB, EC 1.8.99.5), elemental sulfur/polysulfide reduction (DMSO reductases, EC 1.8.5.3; SreABC, no EC), sulfite/tetrathionate reduction (Asr, no EC), thiosulfate reduction (PhsA, EC 1.8.5.5), arsenate reduction (ArrA, EC 1.20.99.1), or methane production (McrA, EC 2.8.4.1). Literature related to the most closely affiliated strains with a characterized strain (when a closely related strain was available) was used to increase confidence in whether the organisms in question were likely to be autotrophic or heterotrophic and likely capable of usage of various electron donors and electron acceptors. This information was used to assign OTUs to functional guilds.

### Phylogenetic analysis

To identify the most closely related organism with a genome sequence available for phylogenetic analyses, we subjected the beta subunit of RNA polymerase (RpoB) or the alpha subunit of DNA gyrase (GyrA) from a representative MAG (lowest % contamination and highest % completeness) to BLASTp analysis against the NCBI nonredundant protein database. The MAG or genome with a corresponding homolog that was most closely related to the GSL MAG of interest was then used as the reference genome to calculate whole-genome pair-wise amino acid identities (AAI). Pair-wise AAI was calculated between the reference and comparator genomes using the Kostas Lab calculator (http://enve-omics.ce.gatech.edu/aai/).

To reconstruct the phylogenomic relationships of the *Ca. Bipolaricaulota* and *Thermoplasmatota* affiliated MAGs, the MarkerFinder program (v.1.1) was employed to detect homologs of 40 universal single-copy housekeeping phylogenetic marker genes. In addition, publicly accessible *Ca. Bipolaricaulota* and *Thermoplasmatota* genomic data from the NCBI and the Genome Tree Database (GTDB) were incorporated into the analysis. The individual proteins encoded by the marker genes were aligned using Clustal Omega (v.1.2.4) (Sievers and Higgins 2018). Subsequently, the concatenated alignment was subjected to maximum likelihood (ML) phylogenetic analysis, employing IQ-TREE (v.1.6.11), with the optimal amino acid substitution model (LG+F+I+R4) among 168 potential models specified. The Bayesian information criterion as implemented in the model testing “TEST” function of IQ-TREE was used. To ensure robustness, ten independent phylogenetic analysis runs were executed and compared. The final ML reconstruction, yielding the most accurate representation, was selected as the definitive phylogenetic tree. The support for the branches in the tree was evaluated by performing 1000 ultrafast bootstraps.

## Results and discussion

### Sediment porewater geochemistry, mineralogy, and microbial activities

**Figure 2.**
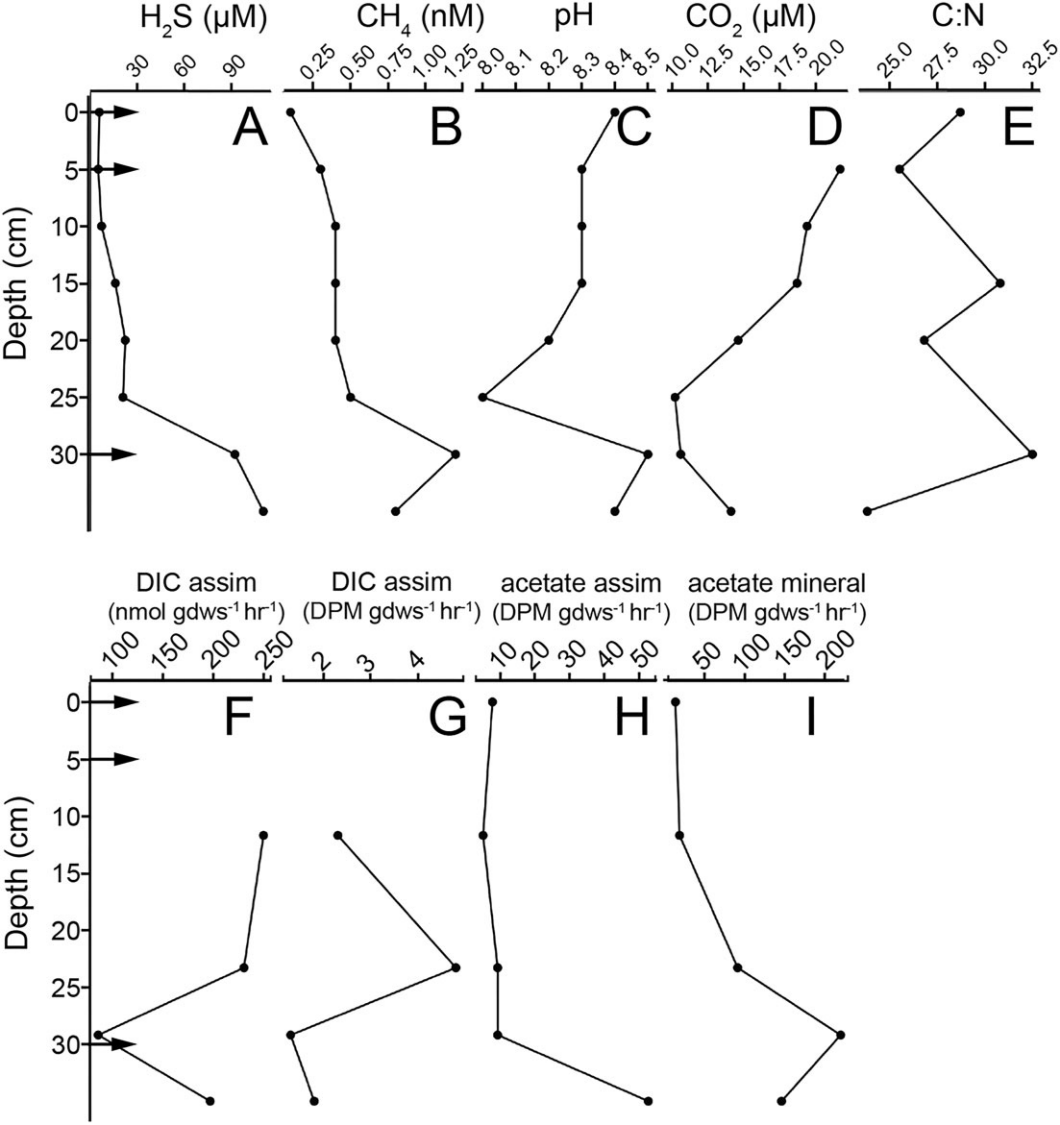
Depth profiles of select geochemical and microbiological measurements from sediment column collected from the NA of GSL. Data adapted from Dunham et al. (2020) with arrows indicating depths utilized for metagenomic analysis. Standing concentrations of relevant chemicals in the sediment column (A) hydrogen sulfide, (B) methane, (C) pH, (D) carbon dioxide, and (E) total organic carbon:total organic nitrogen ratios. Rates of microbial (F) DIC assimilation (nmol gdws^−1^ h^−1^), (G) DIC assimilation (DPM gdws ^−1^ h^−1^), (H) acetate assimilation (DPM gdws ^−1^ h^−1^), and (I) acetate mineralization (DPM gdws ^−1^ h^−1^). Abbreviations: assim: assimilation, DIC: dissolved inorganic carbon, nmol: nanomolar, gdws: gram dry-weight sediment, and DPM: disintegrations per minute.

Trends in the porewater geochemistry and minerology of the NA sediment column from GSL were described previously (Dunham et al. 2020) and select parameters are reintroduced here to provide context for metagenomic data. Porewater total dissolved sulfide increased with depth from 6 µM at the sediment–water interface to 110 µM at a depth of 35 cm (Fig. 2A) and porewater pH decreased in the NA between 0 and 30 cm in depth and then increased markedly at 35 and 40 cm (Fig. 2C). Porewater dissolved CH_4_ concentrations were uniformly low along the depth transect and never exceeded 2 nM (Fig. 2B) while porewater CO_2_ generally decreased with depth (Fig. 2D). The total organic carbon (C) to nitrogen (N) ratio (C:N) in sediments from the NA core fluctuated between 24 and 32 throughout and did not follow an obvious pattern with depth (Fig. 2E). Nonetheless, such high ratios in other lakes have been suggested to reflect input of terrestrial plant matter (Prahl et al. 1994) or could be indicative of substantial processing of algal or Cyanobacterial biomass (C:N ratio of ∼6–7; Redfield 1934), which tends to deplete the ratio. Porewaters were salt saturated (precipitated halite was detected throughout the column) and for simplicity their salinity was assumed to be the same as the waters overlying the sediments (>26%) (data not shown).

Several microbial activities in sediment associated microbial populations were also measured previously (Dunham et al. 2020) and are rerepresented here to provide additional context for metagenomic data. This included DIC assimilation and acetate assimilation and mineralization. Importantly, in the case of acetate assimilation/mineralization, these are presented as disintegrations per minute (DPM) gdws per hour since the amount of added radiolabeled substrate was kept constant but was not normalized to the concentration of native (unlabeled) substrates since these were not measured. Rates of DIC assimilation are presented as both DPM gdws^−1^ h^−1^ and as nmol gdws^−1^ h^−1^ following normalization to total DIC, as described below. Further, these activities were measured on a core that was not frozen on site (unlike that used for molecular and geochemical analyses) and as such, the sediments compacted during transport and do not perfectly align depth-wise with the molecular and geochemical data collected on the other column. Nonetheless, DIC assimilation attributable to microbial cells (primary production) was detected in communities associated with sediments throughout the column and did not vary significantly with depth (Fig. 2G). Acetate assimilation and mineralization rates attributable to biology (secondary production) were also detected in communities associated with sediments (Fig. 2H and I). Rates were lowest at the sediment–water interface and increased with depth by several orders of magnitude, with the highest rate of acetate mineralization detected at a depth of 29.2 cm and the highest rate of acetate assimilation measured at a depth of 35.0 cm. NA sediments exhibited positive relationships between acetate mineralization/assimilation and depth (Pearson *R* = 0.85 and 0.64, respectively).

Rates of DIC assimilation (measured in DPMs) based on assimilation of ^14^C-bicarbonate were converted to rates of total DIC uptake by estimating the concentration of HCO_3_^−^ from dissolved CO_2_ and pH data (see the section “Materials and methods”). When converted, the rates of DIC assimilation ranged from ∼7 to 20 nmol C gdws^−1^ h^−1^ and these also did not follow a trend with depth. Despite the NaCl saturated nature of the sediment core, these rates were within the range of those observed in other freshwater and marine sediments. For example, rates of dark DIC assimilation in four intertidal sediment cores collected from the Eastern Scheldt estuary, the Netherlands, ranged from 0.18 to 7.2 µmol C per cm^3^ day^−1^ near the water-sediment interface only to drop to ∼ 0.02 µmol C per cm^3^ day^−1^ or lower at depths of 2 cm or more (Boschker et al. 2014). Likewise, rates of dark DIC assimilation in intertidal sediments from the German Wadden Sea ranged up to 0.1 µmol C per cm^3^ day^−1^ near the water-sediment interface only to drop to <0.01 µmol DIC per cm^3^ day^−1^ or lower at depths (Lenk et al. 2011). Assuming an average density of sand rich sediment of 2.0 g cm^−3^ (typical range of 1.7–2.3 g cm^−3^; Manger 1963) and converting these to an hourly rate, they become 3.8–146 nmol C gdws^−1^ h^−1^ and 2.1–2.4 nmol C gdws^−1^ h^−1^ in the upper sediments from the Eastern Scheldt estuary and German Wadden Sea, respectively. In the lower sediments of these columns, rates drop to 0.06 (<2 cm depth Eastern Scheldt estuary core) and 0.41 nmol C gdws^−1^ h^−1^ (10 cm depth German Wadden Sea core). In other extreme environments, such as hot spring sediments or proglacial sediments, rates of dark DIC uptake range from 10 to 100 nmol C gdws^−1^ h^−1^ and 0.08 to 0.11 nmol C gdws^−1^ h^−1^, respectively (Urschel et al. 2015, Dunham et al. 2021). Thus, the rates of DIC fixation estimated for the GSL NA sediment core are within the range observed for other aquatic sediments and other extreme environments.

### Taxonomic composition of the sediment core

**Figure 3.**
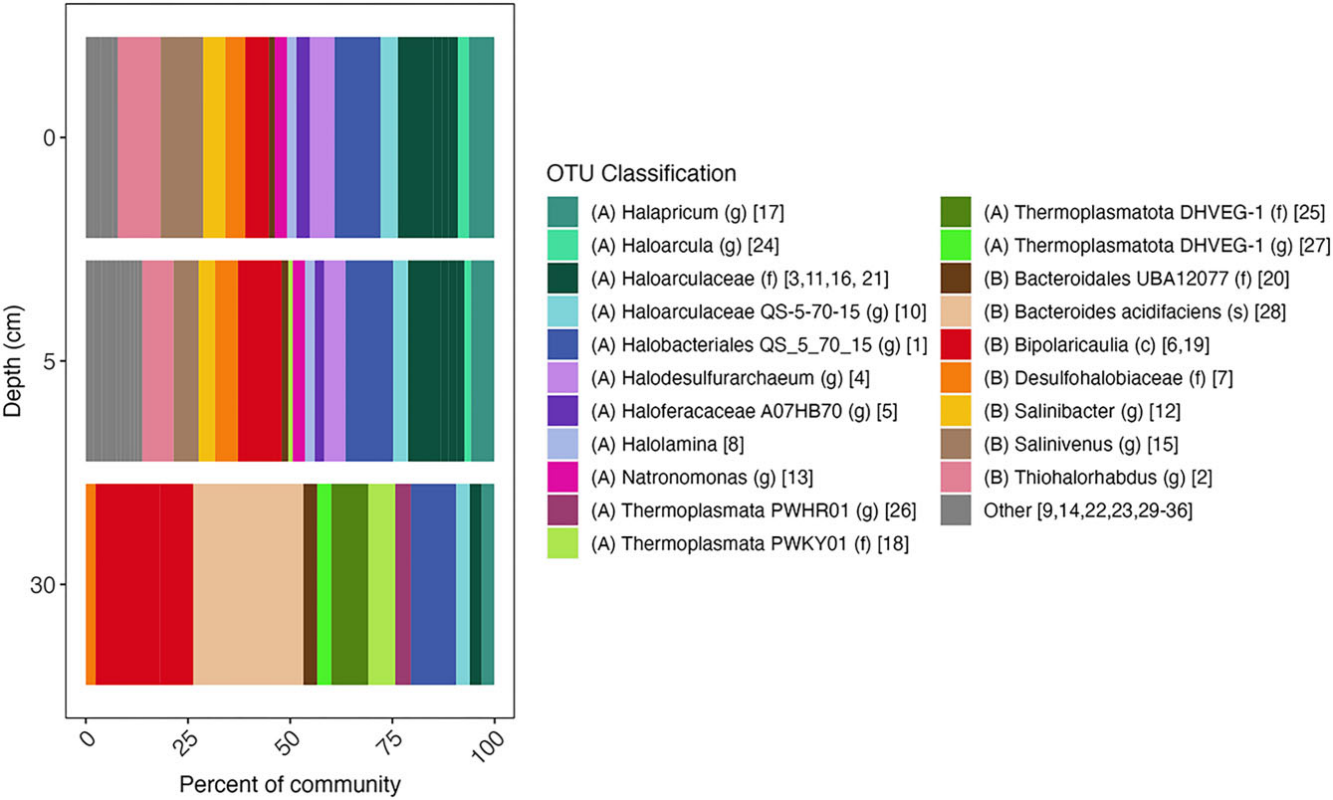
Taxonomic composition of MAGs recovered from depths 0, 5, and 30 cm in a sediment column from the NA of GSL. MAGs were compiled into OTUs using an ANI of >95%. OTUs that were >2% relative abundance of total reads mapped to MAGs are shown; all others are in the “other” category. The domain of each OTU is denoted as (A) for Archaea and (B) for Bacteria. OTUs were classified to the highest taxonomic rank using GTDB-Tk and this is indicated by abbreviations: (c) class level classification, (f) family level classification, (g) genus level classification, and (s) species level classification. OTU designations are indicated in brackets and full taxonomies are reported in Supplemental Table S1.

The taxonomy (Fig. 3; Supplementary Tables S2 and S3), functional potential (Supplementary Table S4), and % identity to organisms with available genomes (Supplementary Table S5) of OTUs that comprised greater than 5% of the mapped reads within at least one depth were analyzed. Additional details of metabolic reconstructions of each OTU are presented in the Supplemental Information. At the 0 cm depth interval, OTUs were affiliated with the archaeal order *Halobacteriales* (OTUs 1, 3, 4, and 17), the bacterial family *Salinibacteraceae* (OTUs 15 and 12), the bacterial genus *Thiohalorhabdus* (OTU 2), and the bacterial class *Ca. Bipolaricaulia* (OTU 6) (Fig. 3; Supplementary Tables S2, S3, and S5). The *Halobacteriales* affiliated OTUs were all predicted to be aerobic heterotrophs based on genome reconstructions (Supplementary Table S4). The presence of putative aerobic and heterotrophic *Halobacteriales* at 0 cm depth in the hypersaline GSL environment is unsurprising, as they have been detected and/or cultivated from GSL before (Post 1977, Baxter 2018, Kemp et al. 2018) and characterized members of this order have been shown to be obligately halophilic, aerobic heterotrophs (Oren 2006). OTUs 12 and 15 were affiliated with family *Salinibacteraceae*, with OTU 12 most closely affiliated with the genus *Salinibacter* and OTU 15 most closely affiliated with the genus *Salinivenus*. Both OTUs are predicted to be aerobic heterotrophs, consistent with the presence of aerobic heterotrophic members of *Salinibacteraceae* in hypersaline lakes (Oren 2019, Viver et al. 2023), including GSL (Almeida-Dalmet et al. 2015, Kemp et al. 2018). The oxygenated and productive waters overlying the sediments of the NA of GSL (Pace et al. 2016, Lindsay et al. 2017) would facilitate the presence of aerobic heterotrophs in surface sediments.

MAGs corresponding to OTU 2 (Fig. 3; Supplementary Tables S2 and S3), which is closely affiliated with the bacterial genus *Thiohalorhabdus* (Supplementary Table S5), encoded homologs of glycolytic pathways, gluconeogenic, and TCA cycle proteins as well as homologs of phosphorubokinase and RuBisCO (Supplementary Table S4), suggesting they correspond to facultative autotrophs. Additionally, OTU 2 encoded homologs of a group 1a [NiFe]-hydrogenase predicted to be involved in H_2_ oxidation (Søndergaard et al. 2016), SreABC involved in anaerobic elemental sulfur (S^0^) reduction (Laska et al. 2003), SoxAB involved in thiosulfate (S_2_O_3_^2−^) oxidation (Wodara et al. 1997), Sqr involved in sulfide (HS^−^) oxidation (Shahak and Hauska 2008), and Cox involved in O_2_ respiration. This is consistent with this OTU being capable of coupling oxidation of H_2_, HS^−^, or S_2_O_3_^2−^ to the reduction of O_2_ or S^0^ to provide energy for autotrophy. The presence of this OTU in surface sediments is consistent with decreased concentrations of H_2_S nearer to the surface of sediment column (Fig. 2A). The genomic characterization of OTU 2 is also consistent with other characterization of this genus, which comprises halophilic, facultatively anaerobic autotrophs that derive energy for carbon fixation primarily through sulfur oxidation (Sorokin et al. 2008).

**Figure 4.**
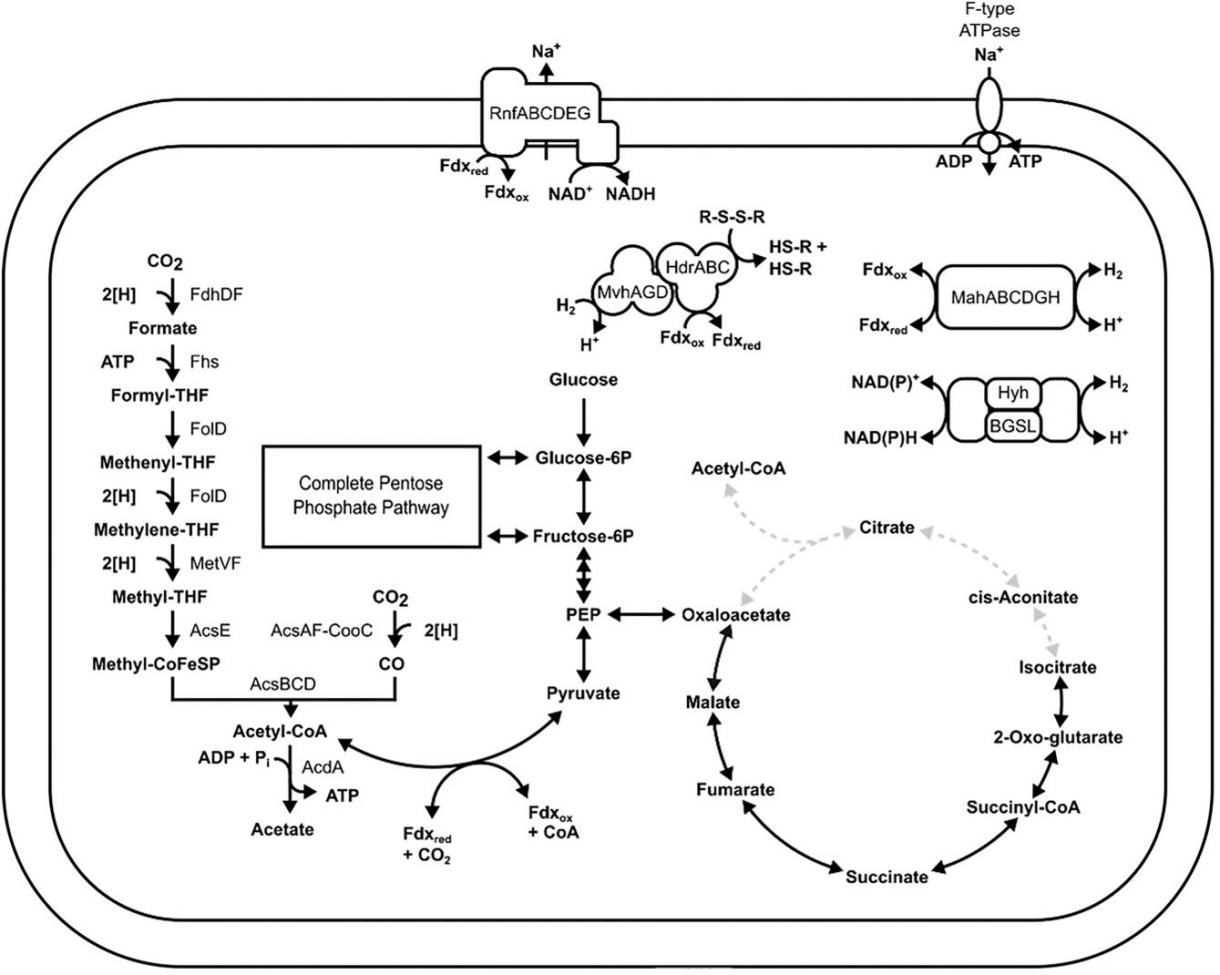
Integrated metabolic models of acetogens including those affiliated with *Ca. Bipolaricaulia* (OTUs_6 and 19; A) and *Thermoplasmatota* (OTUs 18 and 26; B). Abbreviations: FdhDF, formate dehydrogenase; Fhs, formyltetrahydrofolate synthetase; FolD, methylenetetrahydrofolate dehydrogenase; MetVF, methylenetetrahydrofolate reductase; AcsE, 5-methyltetrahydrofolate corrinoid/iron sulfur protein methyltransferase; AcsBCD, acetyl-CoA synthase enzyme complex; RnfABCDEF, Rnf complex; HdrABC, heterodisulfide reductase complex; Hys, [NiFe] group 1a hydrogenase; MvhAGD, [NiFe] group 3c hydrogenase; MahABCDGH, [NiFe] group 4g-hydrogenase; EchABCDEG, energy-converting [NiFe]-hydrogenase complex; and MtrA/MtrH, methyltetrahydromethanopterin: coenzyme M methyltransferase complex subunits A and H.

The *Ca. Bipolaricaulia* affiliated OTU 6 (Supplementary Table S5) is predicted to be anaerobic and to be a hydrogenotrophic acetogen (Fig. 4; Supplementary Table S4; discussed more below). The presence of an obligately anaerobic acetogen in a putatively oxygenated environment (surface sediment) is potentially surprising. However, it is suggested that the presence of abundant aerobic heterotrophs in this depth interval may represent a strong enough sink for O_2_, facilitating the presence of this putative anaerobic acetogen.

At the 5 cm depth, the composition of the community did not change substantially relative to the 0 cm depth, although the relative abundances of several OTUs changed (Fig. 3; Supplementary Tables S2 and S3). OTU 7, most closely affiliated with the bacterial order *Desulfohalobiaceae* (Supplementary Table S5), was found at its highest in abundance at the 5 cm depth interval and is likely an O_2_-tolerant anaerobe (based on identification of Cyd homologs) and facultative autotroph (based on detection of homologs of enzymes involved in the WL pathway; Supplementary Table S4), which is typical for other organisms of this order (Kuever 2014). MAGs affiliated with OTU 7 encode a homolog of a group 1c [NiFe]-hydrogenase predicted to be involved in H_2_ oxidation (Søndergaard et al. 2016) and homologs of Sat, Aps, and DsrAB. This suggests an ability to couple oxidation of H_2_ to reduction of SO_4_^2−^ to generate energy for autotrophy, which is consistent with characterizations of other members of this order (Mussmann et al. 2005, Meyer and Kuever 2007, Santos et al. 2015).

Members of the order *Halobacteriales* (OTUs 1, 3, 4, and 17) generally decreased in relative abundance at the 5 cm depth interval (Fig. 3; Supplementary Tables S2 and S3). Similarly, the abundance of OTUs affiliated with the family *Salinibacteraceae* (OTUs 15 and 12) sharply decreased at the 5 cm depth interval and the abundance of *Salinibacter* (OTU 12) represented less than 5% of community. OTU 2, closely affiliated with the genus *Thiohalorhabdus* (Supplementary Table S5), also decreased in the 5 cm depth interval. In contrast, members of the class *Ca. Bipolaricaulia* (OTU 6) increased in abundance and a second OTU that is also closely affiliated with *Ca. Bipolaricaulia* (OTU 19) was detected in the 5 cm depth interval. OTU 19 encoded a suite of proteins that were similar to those encoded by OTU 6 and, as such, was also classified as an anaerobic hydrogenotrophic autotroph, or more specifically, an acetogen (Fig. 4; Supplementary Table S4; described in more detail below). The decreased abundance of aerobic heterotrophs and increased abundance of anaerobic autotrophs is consistent with an increase in the concentration of HS^−^ with depth (Fig. 2A). The decrease in aerobic heterotrophs is also consistent with the likely decrease of O_2_ in the sediment column (Pace et al. 2016). This, in turn, potentially facilitates the increase in anaerobic autotrophs.

Relative to the 0 and 5 cm depth, the composition of the community shifted substantially in the 30 cm depth sediment interval (Fig. 3; Supplementary Tables S2 and S3). OTUs 1, 6, 18, 19, 25, and 29 were the only OTUs that comprised at least 5% of mapped reads in sediments at the 30 cm depth. Two of these OTUs were affiliated with the class *Ca. Bipolaricaulia* (OTUs 6 and 19), two were affiliated with the phylum *Thermoplasmatota* (OTUs 18 and 25), one was affiliated with the order *Halobacteriales* (OTU 1), and one with the *Bacteroidetes* (OTU 28) (Supplementary Table S5). Of the OTUs present at the 30 cm depth, only OTUs 18, 19, 25, and 28 were not detected at elevated abundances (>5% of total mapped reads) at shallower depths in the sediment column. Notably, OTU 28 (*Bacteroides acidifaciens*) was the most abundant (26.9%) at the 30 cm depth column followed by OTU 6 (15.7%). Of the two OTUs affiliated with the phylum *Thermoplasmatota*, OTU 18 was identified as being part of the order PWKY01, an alphanumeric placeholder taxonomic designation (Rinke et al. 2021), and OTU 25 was identified as being a member of the Deep Hydrothermal Vent Euryarchaeal Group 1 (DHVEG-1; Supplementary Table S5), which has recently been proposed to be renamed *Thermoprofundales* (Zhou et al. 2019). As described in more detail below, both OTUs 18 and 25 are predicted to be anaerobic and facultatively autotrophic.

### Unique putatively autotrophic OTUs

**Figure 5.**
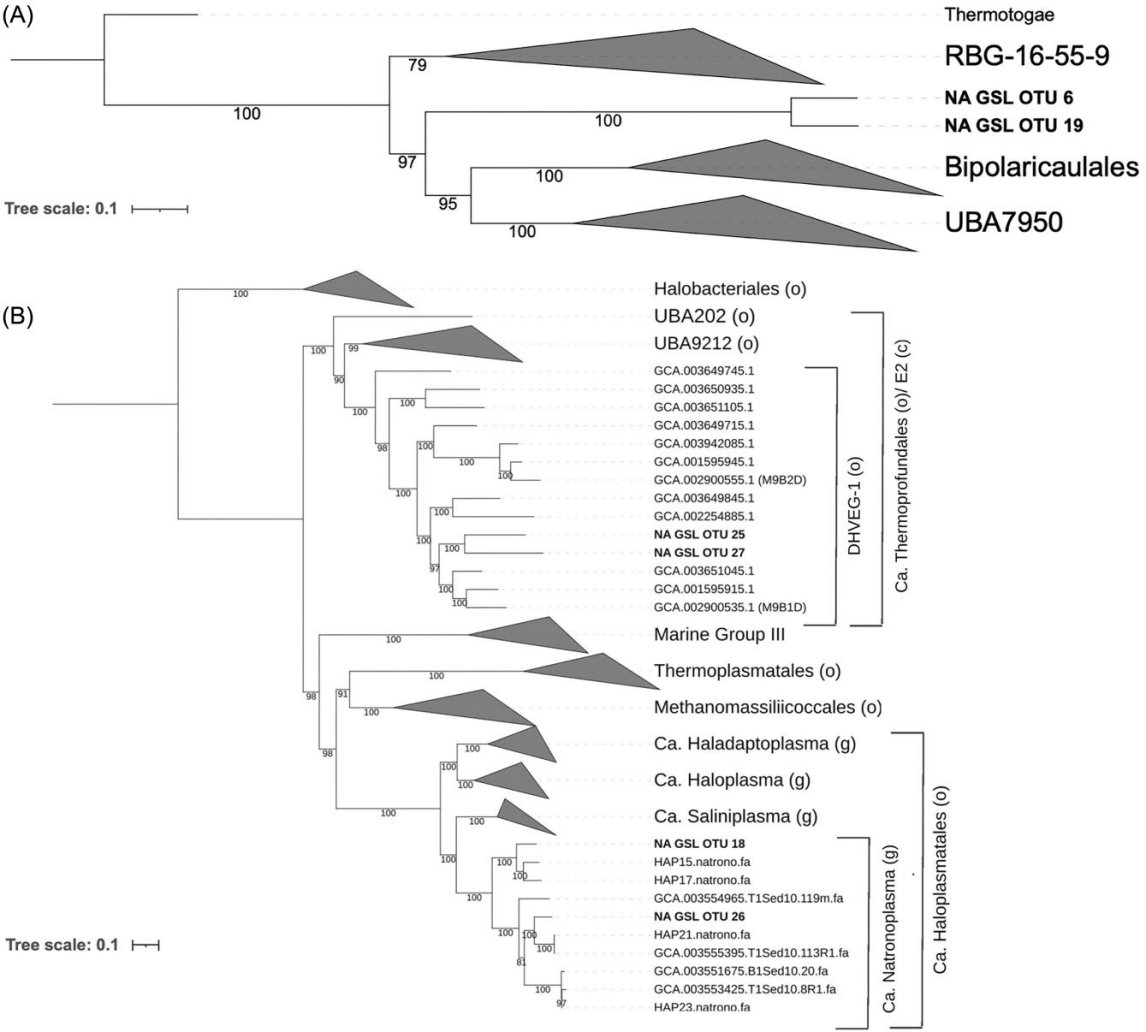
Phylogenomic reconstruction of putative select OTUs in a sediment column collected from the NA of GSL. (A). Phylogeny of *Ca. Bipolaricaulia* affiliated OTUs 6 and 19 in relation to closely related taxonomic lineages. Genomes from the representative *Thermotogota* (*Thermotoga maritima* MSB8, *Fervidobacterium pennivorans* DSM 9078, and *Pseudothermotoga thermarum* DSM 5069) were used as the outgroup. (B) Phylogeny of *Thermoplasmatota* affiliated OTUs 18, 25, 26, and 27 in relation to closely related taxonomic lineages. Genomes from the order *Halobacteriales* were used as the outgroup. Representative sequences (M9B1D and M912D) from archaeal E2 order (proposed *Candidatus Thermoprofundales*) are bolded. Each branch of the tree displays bootstrap support values (out of 1000 bootstraps).

OTUs not previously described in GSL or that encode putative autotrophic pathways were subjected to additional phylogenetic analysis to determine their relationships to other MAGs or genomes (Fig. 5). The metabolisms of these OTUs were further scrutinized to identify potential electron donors and acceptors that would provide energy to drive CO_2_ fixation or that would inform on their overall mode of metabolism (if not putatively autotrophic) (Supplementary Table S4). This included members of the *Ca. Bipolaricaulia* (Fig. 4) and *Thermoplasmatota* OTUs (described in text).

#### *Candidatus Bipolaricaulia* (OTUs 6 and 19)

A ML phylogeny was constructed to better depict the relationship of the two *Ca. Bipolaricaulia*-affiliated OTUs (GBDK taxonomic classification) from the NA sediment column in GSL to those identified previously (Fig. 5A). The two *Ca. Bipolaricaulia* OTUs branched basal to MAGs affiliated with the class *Ca. Bipolaricaulia* and its sister lineage UBA7950. The GSL OTUs formed a monophyletic lineage between existing members of the *Ca. Bipolaricaulia* and those that form the clade RBG16-55-12, that along with clade UBA1414 (not included in phylogeny), were formerly known as “OPB41” (Hugenholtz et al. 1998). AAI scores indicate these OTUs are most closely related to single cell genomes corresponding to members of this clade (Merino et al. 2020). These cells, like many members of the *Ca. Bipolaricaulia* (Youssef et al. 2019, Colman et al. 2022), are acetogens that fix carbon via the WL pathway. *Candidatus Bipolaricaulia* MAGs previously identified from halite crusts in the BSL (McGonigle et al. 2022a), cluster within the *Ca. Bipolaricaulia* but are phylogenetically distinct from those identified in the NA of the GSL.

Phylogenetic placement of the GSL OTUs 6 and 19 between RBG-16–55–9 and *Ca. Bipolaricaulales*/UPA7950 lineages (Fig. 5A), whose members often encode the WL pathway and that are described as acetogens (Youssef et al. 2019, Colman et al. 2022, McGonigle et al. 2022a), suggests they may also be anaerobic autotrophs and potentially acetogenic. Indeed, metabolic reconstruction of OTUs 6 and 19 revealed homologs of all proteins involved in the WL pathway for CO_2_ fixation (Fig. 4; Supplementary Table S4). These OTUs did not encode homologs of other terminal oxidases, suggesting they are likely anaerobic autotrophs. Further, these OTUs encoded homologs of four [NiFe]-hydrogenase complexes classified in groups 1a, 3b, 3c, and 4 g (Søndergaard et al. 2016). Group 1a hydrogenase (HysAB) is positioned adjacent to the methylene-tetrahydrofolate (MTHF) reductase subunits MetVF in the genome, suggesting it may be involved in providing electrons for MTHF reduction. The bidirectional group 3b hydrogenase, HyhBGSL, is predicted to allow for reversible NAD(P)^+^ reduction with H_2_, while the group 3c hydrogenase MvhAGD is predicted to form a complex with HdrABC and bifurcate electrons from H_2_ oxidation for simultaneous reduction of ferredoxin (Fd) and heterodisulfide (Kaster et al. 2011). The group 4 g hydrogenase, MahABCDGH, is colocalized on a contig with ion transporter and antiporter genes. This complex may function in an anapleurotic role by balancing the ratio of oxidized to reduced Fd and/or ion balance (Lie et al. 2012). Both OTUs also encode homologs of the Rnf complex, RnfABCDEG. Rnf links the Fd and NADH pools with the proton/sodium ion motive force. When the concentration of Fd is greater than NAD^+^, electron flow is to NAD^+^ and this is coupled to ion translocation out of the cell, conserving energy (Westphal et al. 2018). When the concentration of NADH is greater than Fd, Rnf works in reverse. The Rnf complex plays an indispensable role in the energy metabolism of anaerobes as it maintains the ion gradient across the membrane. This gradient, in turn, allows for ATP synthesis via an F type ATP synthase. Importantly, GSL OTUs encode complete pentose phosphate and glycolytic pathways as well as a nearly complete TCA cycle, suggesting the possibility that these organisms can possibly ferment as well. As such, these OTUs are designated as anaerobic facultative autotrophs that are likely capable of hydrogenotrophic acetogenesis and fermentation.

#### Thermoplasmatota (OTUs 18, 25, 26, and 27)

A ML phylogeny was constructed to better depict the relationship of the four *Thermoplasmatota*-affiliated OTUs from the NA sediment column in GSL to those identified previously (Fig. 5B). Reference genomes from the archaeal E2 order (GTDB alphanumeric classification) and recently published genomes corresponding to the proposed archaeal orders *Candidatus Haloplasmatales* (Zhou et al. 2022) and *Candidatus Thermoprofundales* (Zhou et al. 2019) are also included. Two of the OTUs (OTUs 25 and 27) from GSL were taxonomically identified at the lowest rank to the archaeal class E2 and order/family DHVEG-1 (GTDB classification). Phylogenetic analyses of these OTUs indicate that they branch within the clade comprising members of the E2 class, and more distinctly, between two of the major reference MAGs from the proposed *Ca. Thermoprofundales* order (MAGs M9B1D and M912D) (Zhou et al. 2022). These MAGs are referred to as *Thermoprofundales* for the remainder of this paper.

Phylogenetic reconstruction of OTUs 18 and 26 affiliated with the archaeal family PWKY01 (GBDK classification) shows they both cluster within the proposed genus *Candidatus Natronoplasma*, one of four genera within the proposed order *Ca. Haloplasmatales* (Zhou et al. 2022). Consistent with this designation, both OTUs 18 and 26 share >65% AAI with the proposed genomes that comprise *Ca. Natronoplasma* (Supplementary Fig. S1), which is above the cutoff typically used to designate new genera. Therefore, it is likely that these two OTUs represent new species within this genus. These OTUs are referred to as *Ca. Natronoplasma* herein.

The two GSL *Thermoprofundales* OTUs (OTUs 25 and 27) encoded nearly complete glycolytic and archaeal pentose phosphate pathways (Supplementary Table S4). These OTUs encoded homologs of [NiFe]-hydrogenases classified as groups 3b, 3c, and 4d (Søndergaard et al. 2016). The group 3b homolog is predicted to reversibly couple H_2_ oxidation to reduction of NADP^+^, the group 3c homolog is predicted to form a complex with Hdr and bifurcate electrons from H_2_ oxidation to the simultaneous reduction of Fd and heterodisulfide, while the group 4d homolog is predicted to couple H_2_ oxidation to Fd reduction accompanied by ion translocation. The only terminal oxidase identified in MAGs corresponding to these OTUs was a homolog of a putative fumarate reductase flavoprotein, indicating these OTUs are anaerobes. OTU 25 (but not OTU 27) encoded homologs of proteins involved in the reverse TCA cycle (rTCA), including ATP citrate lyase (AclA), ATP citrate synthetase (CcsA), and citryl-CoA synthetase (CcsB). The MAGs corresponding to OTUs 25 and 27 have similar completeness at 87.9% and 85.7%, respectively, and the lack of rTCA homologs encoded in OTU 27 may allow cohabitation by minimizing niche overlap. A previous study of *Thermoprofundales*-affiliated MAGs recovered from brine pools in the Red Sea identified numerous homologs of [NiFe]-hydrogenase and a homolog of fumarate reductase (Mwirichia et al. 2016). However, unlike OTU 25, the Red Sea MAGs encoded the WL pathway. Neither OTU 25 nor OTU 27 encode a sufficient number of proteins that would suggest that they can fix carbon via the WL pathway. As such, OTU 25 is designated as an anaerobic, facultatively hydrogenotrophic autotroph, which is consistent with previous studies of closely related members of the *Thermoprofundales* (Mwirichia et al. 2016), whereas OTU 27 is designated as a facultative anaerobic heterotroph.

OTUs 18 and 26 both encode nearly complete glycolytic pathways (Supplementary Table S4). Neither OTU encoded homologs of terminal oxidases, indicating they are anaerobic and likely fermentative. The GSL OTUs encode homologs of group 3c and 4d [NiFe]-hydrogenases (Søndergaard et al. 2016). Group 3c [NiFe]-hydrogenases (Mvh) are predicted to reversibly link H_2_ oxidation to the simultaneous reduction of Fd and heterodisulfide while group 4d [NiFe] hydrogenase (Ech) is predicted to form a complex with H^+^/Na^+^ antiporters that couple reversible Fd oxidation to H_2_ production linked to H^+^/Na^+^ translocation across the cell membrane (Peters et al. 2015). It is possible that Ech allows for Fd/H_2_-driven ion transport in OTUs 18 and 26. Without additional data (e.g. more complete genomes and cultivation data), these OTUs are conservatively labeled as fermentative heterotrophs. It is not clear what role the group 3c [NiFe]-hydrogenase would have in this metabolic background.

### Trends in metabolism as a function of depth

**Figure 6.**
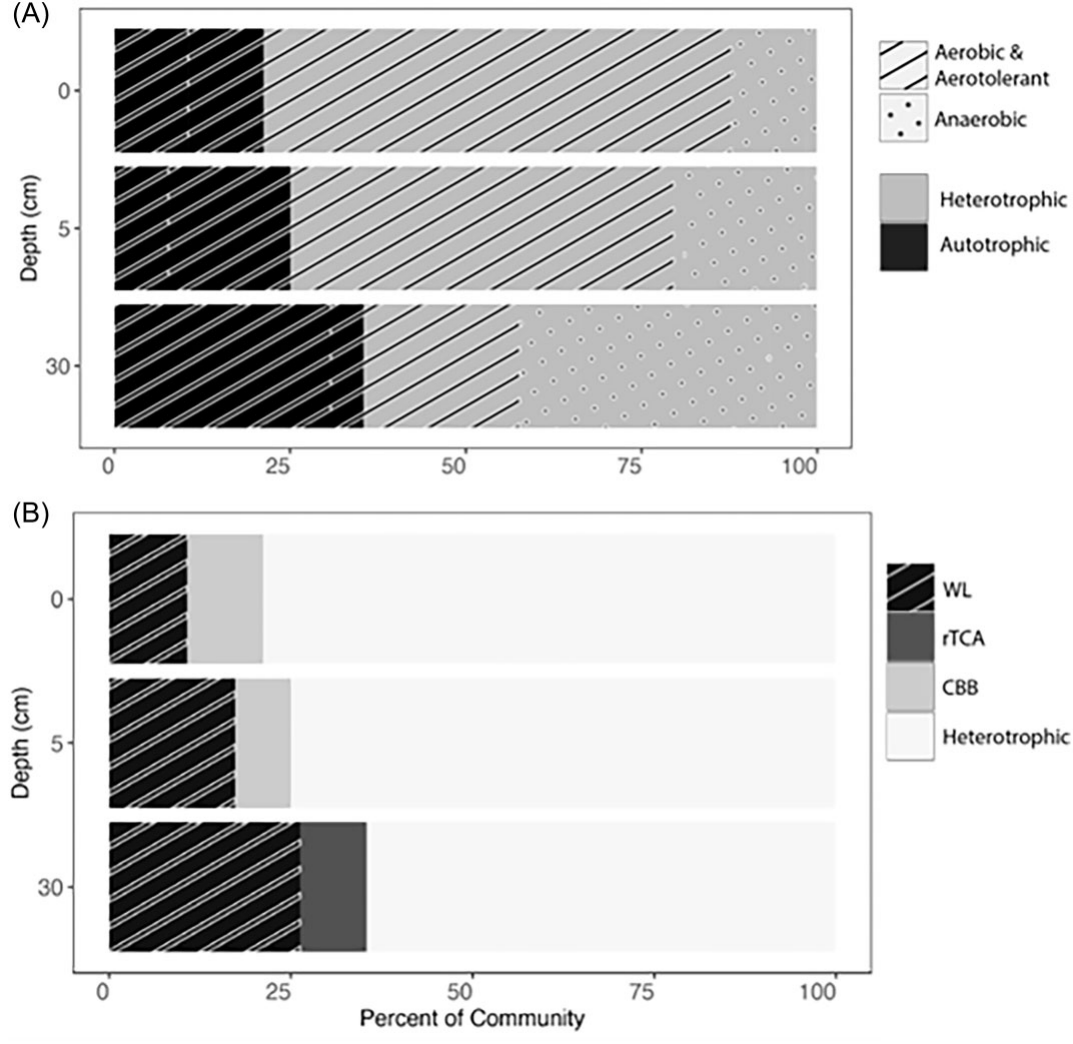
Inferred metabolic potentials of MAGs recovered from 0, 5, and 30 cm depth intervals in a sediment column from the NA of GSL. (A) Carbon source and O_2_ utilization were inferred by first clustering MAGs into OTUs based on 95% ANI and then functionally annotating a MAG representative of each OTU. Relative abundances for each OTU were determined by mapping reads back to each constituent MAG. OTUs encoding autotrophic pathways were classified as autotrophs, while those lacking autotrophic pathways and possessing pathways indicative of heterotrophy (e.g. glycolysis) were classified as heterotrophs. Similarly, OTUs encoding pathways for utilizing O_2_ as a terminal electron acceptor were deemed aerobes, while those with cytochromes capable of O_2_ detoxification were deemed aerotolerant. OTUs lacking homologs of O_2_-metabolizing pathways were deemed anaerobes. The black and gray bars depict the proportions of autotrophs and heterotrophs in the community, respectively. Superimposed hashing depicts the proportions of aerotolerant and aerobic microbes, while the dots depict the proportion of anaerobes. (B) OTUs were further classified based on encoded carbon-fixing pathways. Abbreviations: WL: Wood–Ljungdahl pathway, rTCA: reductive tricarboxylic acid cycle, and CBB: Calvin–Benson–Bassham cycle.

Each of the OTUs were assigned to functional guilds describing their potential to utilize O_2_ (aerobe/aerotolerant, anaerobe) and potential carbon metabolism (heterotroph and autotroph). The abundance of aerobes decreased, and the abundance of anaerobes increased with depth (Fig. 6), likely due to the reduced availability of O_2_ as inferred from the increasing concentrations of sulfide. Similarly, the abundance of heterotrophs decreased with depth while the abundance of autotrophs increased with depth, perhaps due to decreasing availability of quality organic carbon. Interestingly, the fraction of autotrophs that were anaerobic increased with depth, with nearly all the anaerobic OTUs inferred to be obligate anaerobes at the 30 cm depth interval.

Since anaerobes can utilize any of the six known pathways to fix CO_2_, the increased fraction of anaerobic autotrophs at depth prompted an analysis of the predominant carbon-fixation pathways in these organisms. Autotrophs encoding the WL pathway were identified in each of the three depth intervals, and their abundance increased with depth. This was primarily attributed to the increased abundance of obligately anaerobic OTUs affiliated with the *Ca. Bipolaricaulia* and *Desulfohalobiaceae* as a function of depth. In contrast, autotrophs encoding the CBB cycle were identified only in the 0 and 5 cm depth intervals and were affiliated with the genus *Thiohalorhabdus*. An autotroph encoding the rTCA cycle was identified at the 30 cm depth only and was affiliated with the *Thermoprofundales*.

Among the CO_2_-fixing pathways utilized by microorganisms, the WL pathway is considered to be the simplest (fewest enzymes involved), most ancient (Russell and Martin 2004), and the least energetically expensive (Bar-Even et al. 2010, Fuchs 2011). In fact, the pathway is exergonic (Fuchs 1994) and releases enough free energy to drive ATP synthesis if that energy is conserved (Thauer et al. 1977). The WL pathway requires ∼1 ATP for the synthesis of 1 pyruvate from CO_2_ whereas the CBB pathway requires 7 ATP, the rTCA requires 2–3 ATP, the DC/4-HP requires 5 ATP, and the 3-HP/4-HB requires 9 ATP. As such, anaerobic autotrophs operating the WL pathway have been described as being given “a free lunch that they are paid to eat” (Shock et al. 1998).

The WL pathway is the only autotrophic pathway that is exclusively found in anaerobes, likely due to the O_2_ sensitivity of many of its enzymes (Ragsdale and Pierce 2008, Fuchs 2011). The utilization of this lower energy demanding pathway may be consistent with the lower energetic yields of anaerobic metabolisms (Thauer et al. 1977, Bar-Even et al. 2010). Based on the distribution of autotrophic pathways among cultivars that operate low energy yielding metabolisms (anaerobes) or in environments that impose high energy stress, it has been hypothesized that the WL pathway allows cells to fix CO_2_ under conditions that would be otherwise unfavorable (Montoya et al. 2012). The increased prevalence of the WL pathway with increasing depth in the NA GSL sediment column supports this hypothesis, in particular due to the high energetic stress that autotrophs are likely to experience due to polyextremophilic conditions (e.g. hypersalinity and euxinia) that would be increasingly encountered with depth. The identification of diverse and often novel WL pathway encoding anaerobic extremophiles in the euxinic sediments of GSL call for additional efforts to cultivate these organisms for detailed physiological and biochemical study and to further characterize the biodiversity of anaerobic sediments in hypersaline environments. Such efforts will help identify additional adaptations that allow life to thrive under polyextremophilic conditions on Earth and potentially on other planetary bodies.

## Supplementary Material

fiae105_Supplemental_Files
