# Supplementary material for: Wood–Ljungdahl pathway encoding anaerobes facilitate low-cost primary production in hypersaline sediments at Great Salt Lake, Utah
Source: FEMS Microbiol Ecol. 2024 Jul 25;100(8):fiae105. doi: 10.1093/femsec/fiae105 (PMC11287216; doi:10.1093/femsec/fiae105)
Supplement: fiae105_Supplemental_Files [file fiae105_supplemental_files.zip › MB_515_Supp.data_Figs.docx]

**
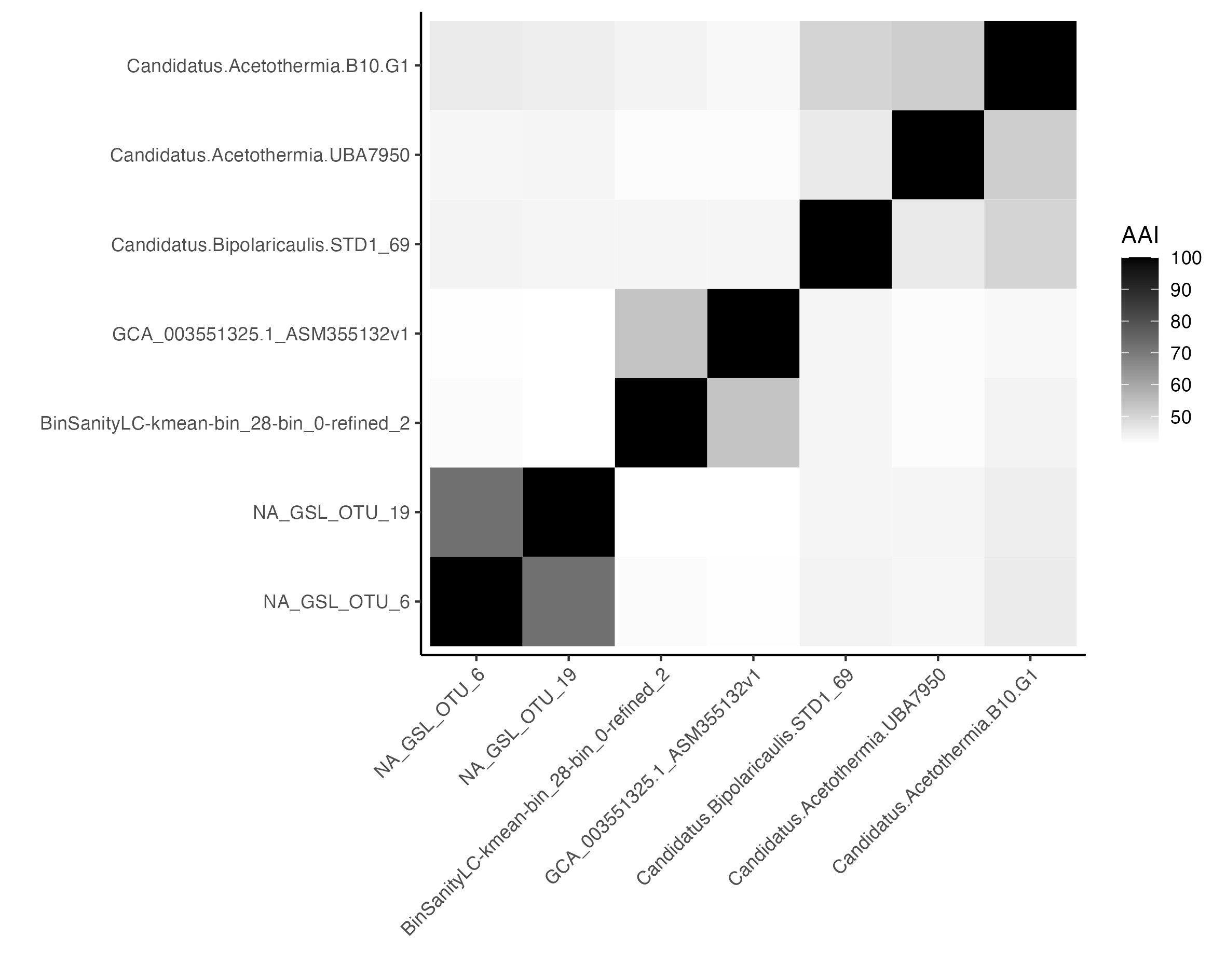
 (a)**


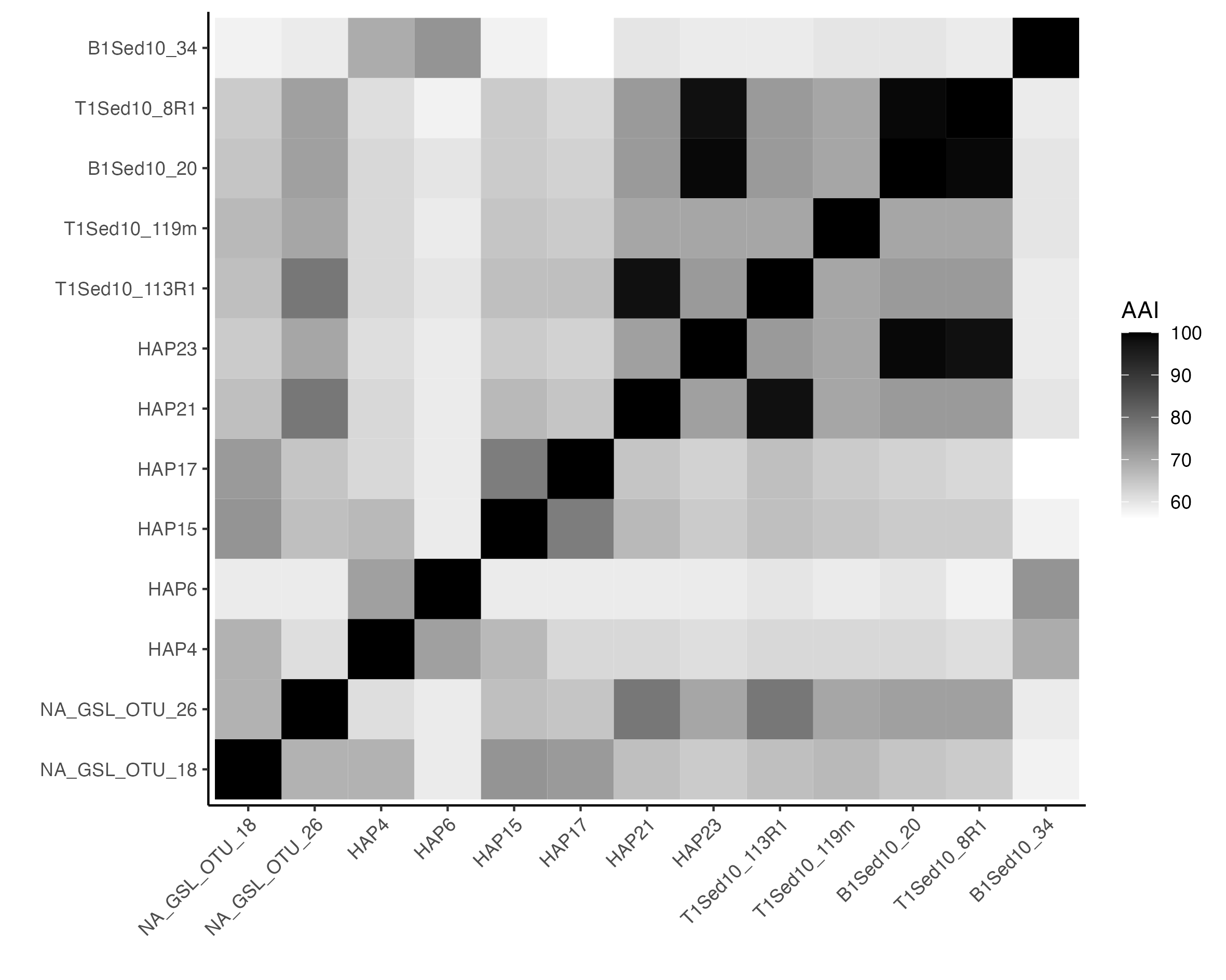


**(b)**

**Supplemental Figure 1:** Heatmaps displaying pairwise average amino acid identity (AAI) between representative metagenomic assembled genomes (MAG) for operational taxonomic units (OTUs) and the genomes of closely related strains. **a**) AAI calculated between OTUs for class *Ca. Bipolaricaulia* (NA_GSL_OTU_6 and NA_GSL_OTU_19) and five reference MAGs.

**b**) AAI calculated between OTUs for phylum *Thermoplasmatota* (NA_GSL_OTU_18 and NA_GSL_OTU_26) and 11 reference MAGs.
