# Supplementary material for: Wood–Ljungdahl pathway encoding anaerobes facilitate low-cost primary production in hypersaline sediments at Great Salt Lake, Utah
Source: FEMS Microbiol Ecol. 2024 Jul 25;100(8):fiae105. doi: 10.1093/femsec/fiae105 (PMC11287216; doi:10.1093/femsec/fiae105)
Supplement: fiae105_Supplemental_Files [file fiae105_supplemental_files.zip › MB_515_Supp.data_Text.docx]

[OTU 1: (Archaea (A)); *Halobacteriales* QS_5_70_15 (genus (g))](#_eu4r1o8uwbfs)

[OTU](#_rgmf8w6j88km)  [2: (Bacteria (B)); *Thiohalorhabdus*](#_rgmf8w6j88km)

[OTU 3: (A); *Haloarculaceae* (family (f))](#_a1fyfm9dmkja)

[OTU 4: (A) *Halodesulfurarchaeum* (g)](#_lyqngjbfb2in)

[OTU 5: (A) *Haloferacaceae* A07HB70 (g)](#_muviapm9hfjv)

[OTU 6: (B) *Ca. Bipolaricaulia* (class (c))](#_y8gxlbqgppfh)

[OTU 7 (B) *Desulfohalobiaceae*](#_ir0b9bhwuirg) (f)

[OTU: 8 *Haloamina*](#_6tz0fr9oalvm) (g)

[OTU 9: (A) *Halovenus* (g)](#_a8ogth8t7ww0)

[OTU 10: (A) *Haloarculaceae* QS-5-70-15](#_dhywoq902534) (g)

[OTU 11: (A) *Haloarculaceae* (f)](#_b7xpiokd2j1l)

[OTU12: (B) *Salinibacter* (g)](#_wov7ro3er80h)

[OTU](#_gc78vwq1jo59) [13: (A) *Natronomonas*](#_gc78vwq1jo59) (g)

[OTU 15: (B) *Salinivenus* (g)](#_juquagbdzdew)

OTU [16: (A) Haloarculaceae (f)](#_xs18kop9e2od)

[OTU 17: (A) *Halapricum*](#_wknnrvf883rx) (g)

[OTU 18: (A) PWKY01 (f)](#_nwj0mzlmkrxp)

[OTU 19: (B) *Ca. Bipolaricaulia* (c)](#_7jo1u2wimlgh)

[OTU 20: (B) UBA12077 (f)](#_w4f97umkodiq)

[OTU 21: (A) *Haloarculaceae* (f)](#_xypy05fy1w8x)

[OTU 22: (A) *Halobacteriales* (o)](#_5tp7w65d77ge)

[OTU 23: (A) *Halovenus* sp004015825 (s)](#_pjnembco9t99)

[OTU 24: (A) *Haloarcula* (g)](#_jmch0yt1da5k)

[OTU 25: (A) DHVEG-1 (f)](#_ul1618rxfe4n)

[OTU 26: (A) PWHR01(g)](#_htq8m8aeg38f)

[OTU 27: (A) DHVEG-1 (f)](#_igswr4igljgq)

[OTU 28: (B) *Bacteroides acidifaciens* (s)](#_sib9gq2ws180)

[OTU 29: (A) *Haloplanus* (g)](#_k4qyl6s4mzqt)

[OTU 30: (B) *Paceibacterales* (order (o)](#_u5w91z4nh7nx))

OTU 31: (A) *Halofilum* (g)

[OTU 32: (B) *Brocadiae* (c)](#_ul6ew4hln47)

[OTU 33: (A) *Haloferacaceae* (f)](#_g62nhjln592b)

[OTU 34: (A) *Natronomonas* (g)](#_n1blqrq0lmes)

[OTU 35: (A) *Nanosalinaceae*](#_mqeizfwqldpg) (f)

[OTU 36: (A) *Halobacteriales*](#_q22605wlyhbh) (o)

#

# OTU 1: (Archaea (A)) *Halobacteriales* QS_5_70_15 (genus (g))

Estimated genome size (5 cm depth) = 3.85 Mbp; completeness = 95.2%; contamination = 3.0%

Relative abundance within community = 11.3% 0 cm, 11.7% 5 cm, and 11.1% at 30 cm

Metagenome assembled genomes (MAGs) corresponding to OTU 1 were identified in all three depths of the sediment column in the North Arm (NA) of Great Salt Lake (GSL). These MAGs do not encode for autotrophic pathways and thus are designated as heterotrophs based on the presence of a complete glycolytic and tricarboxylic acid (TCA) cycles. MAGs encoded homologs of the *caa3*-type cytochrome c oxidase (CoxAB), indicating an ability to respire oxygen (O_2_). Homologs of other terminal oxidases were not identified. MAGs also encoded homologs of sulfur dioxygenase (Sdo), which have been previously shown in *Erythrobacter flavus* to be involved in thiosulfate oxidation (Zhang et al., 2020) and in *Acidithiobacillus* to be involved in sulfur oxidation (Wu et al., 2017). Yet, a different study that created a Sdo knockout mutant in *Acidithiobacillus* suggests a role in sulfur detoxification (Wang et al., 2014). As such, it is unknown if Sdo contributes to energy metabolism through the oxidation of sulfur compounds or if it is involved in sulfur detoxification. As such, OTU 1 was conservatively designated as an aerobic heterotroph with the possibility of a mixotrophic energy metabolism (oxidation of sulfur compounds to supplement organic carbon as an electron donor).

# OTU 2: Bacteria (B); *Thiohalorhabdus* (g)

Estimated genome size (0 cm depth): 2.88 Mbp; completeness = 98.3%; contamination = 3.0%

Relative abundance within community = 10.5% at 0 cm; 7.7% at 5 cm.

MAGs corresponding to OTU 2 were identified at the 0 cm and 5 cm depths of the sediment column in the NA of GSL. The *Thiohalorhabdus* OTU encoded a complete glycolytic pathway, a complete TCA cycle, and the Calvin cycle. In addition, *Thiohalorhabdus* MAGs encode type *caa3*- and *cbb3*-type cytochrome *c* oxidase complexes (CoxAB*,* CcoNOP, respectively). Homologs of other terminal oxidases were not identified. The GSL *Thiohalorhabdus* MAG encodes homologs enabling sulfide oxidation (FccB, Sqr), sulfur oxidation (Sdo, Sor), and thiosulfate oxidation (Sox). The OTU is suggested to be a facultative autotroph, with energy derived from aerobic oxidation of sulfur compounds (sulfide, thiosulfate, and/or elemental sulfur) or aerobic organic carbon respiration.

#

# OTU 3: (A) *Haloarculaceae* (f)

Estimated genome size (0 cm depth) = 2.85 Mbp; completeness = 97.0%; contamination = 3.3%

Relative abundance within community = 8.7% at 0 cm; 8.0% at 5 cm

MAGs corresponding to OTU 3 were identified at the 0 cm and 5 cm depths of the sediment column in the NA of GSL. OTU 3 could only be classified to the family level and is affiliated with *Haloarculaceae*. MAGs encode homologs of the *caa3*-type cytochrome c oxidase (CoxAB), suggesting an ability to respire O_2_. No other terminal oxidases were identified. MAGs encode complete glycolytic and gluconeogenic pathways, as well as the TCA cycle and a V/A-type ATP synthase. MAGs encode for molybdenum-dependent carbon monoxide dehydrogenase (CODH) and formate dehydrogenase (FDH), suggesting an ability to oxidize CO and formate, respectively. MAGs also encode for sulfur oxidation (Sdo, Sor), as well as DMSO (MsuD, DdhA, DmoB) and organic sulfur metabolism (AspB, MetY, MetC, MetE, AhcY, MegL, MetK). This indicates the possibility of a mixotrophic energy metabolism, where sulfur compounds are oxidized to supplement organic carbon as an electron donor. Other cultured species of this family (Han & Cui, 2014b; Waino et al., 2000) have diverse aerobic metabolisms, with several able to ferment when O_2_ is not available. Consistent with this, GSL MAGs encode PorA suggesting an ability to ferment pyruvate. As such, OTU 3 is designated as an aerobic heterotroph with the possible ability to ferment and to augment its heterotrophic energy metabolism via oxidation of a variety of sulfur compounds.

#

# OTU 4: (A) *Halodesulfurarchaeum* (g)

Estimated genome size (0 cm depth) = 2.20 Mbp; completeness = 96.3%; contamination = 1.1%

Abundance within community = 6.0% at 0 cm; 5.2% at 5 cm

MAGs corresponding to OTU 4 were identified at the 0 cm and 5 cm depths of the sediment column in the NA of GSL. OTU 4 is closely related to the archaeal genus *Halodesulfurarchaeum*, which has been described as a strictly anaerobic lithoheterotrophic archaeon (Sorokin et al., 2017). However, GSL MAGs do not encode autotrophic pathways but do encode homologs of the *caa3*-type cytochrome *c* oxidase (CoxAB) and cytochrome *bd* ubiquinol oxidase (CydAB), indicating an ability to respire O_2_. The GSL MAGs also encode for complete glycolytic, pentose phosphate, and TCA cycles, as well as a V/A-type ATP synthase. Further, MAGs encode polysaccharide degradation pathways, including mannan endo-1,4-beta-mannosidase, which degrades endohemicellulases. MAGs encode molybdenum-dependent carbon monoxide dehydrogenase (CODH) as well as formate dehydrogenase (FDH), suggesting an ability to oxidize CO and formate, respectively. MAGs encode for sulfur oxidation (Sdo, Sor), DMSO degradation (ddhA), and organic sulfur metabolism (MetB, MetY, MetC, MetE, AchY, MegL, MetK). This indicates the possibility that sulfur compounds are oxidized to supplement organic carbon as an electron donor. GSL MAGs also encode nitrite oxidation (NxrB) and arsenite oxidation (ArxA) capabilities, providing another source of electrons. MAGS encode for the ability to reduce chlorite (Cld) and perchlorate (PcrA, PcrB). Perchlorate, in many bacteria, can act as an electron acceptor (Bardiya & Bae, 2011). This may be an alternative electron acceptor to O_2_. OTU 4 is designated as a facultative anaerobe and a heterotroph with possible mixotrophic energy metabolism.

# OTU 5: (A) *Haloferacaceae* A07HB70 (g)

Estimated genome size (0 cm depth) = 2.56 Mbp; completeness = 93.3%; contamination = 1.8%

Relative abundance within community = 3.3% at 0 cm; 2.3% at 5 cm

MAGs corresponding to OTU 5 were identified in the 0 cm and 5 cm depths of the sediment column in the NA of GSL. MAGs encode glycolytic and gluconeogenesis pathways, and a partially complete TCA cycle, indicating that OTU 5 is a heterotroph. MAGs also encode molybdenum-dependent carbon monoxide dehydrogenase (CODH) as well as formate dehydrogenase (FDH), suggesting an ability to oxidize CO and formate, respectively. MAGs encode homologs of the *caa3*-type cytochrome *c* oxidase (CoxAB) and cytochrome *bd* ubiquinol oxidase (CydAB), indicating an ability to respire O_2_. Homologs of other terminal oxidases were not identified. MAGs also encode homologs of NDH2, an alternative to NADH dehydrogenase (Complex I) and a V/A-type ATP synthase. Previously characterized members of the *Haloferacaceae* are halophilic and are aerobic heterotrophs (Burns et al., 2007; Oren et al., 1995), consistent with the data presented here. As such, OTU 5 is designated as an aerobic heterotroph.

#

# OTU 6: (B) *Ca. Bipolaricaulia* (class (c))

Estimated genome size (0 cm depth) = 1.78 Mbp; completeness = 88.0%; contamination = 0.0%

Relative abundance within community = 5.8% at 0 cm, 9.0% at 5 cm, and 15.7% at 30 cm

MAGs corresponding to OTU 6 were identified in all depth intervals of the sediment column in the NA of GSL. MAGs encode a complete Wood-Ljungdahl pathway, as well as homologs of [NiFe]-hydrogenases that belong to Groups 1a, 4a, and 4g, based on the HydDB classification tool (Sondergaard et al., 2016). MAGs encoded homologs of PhsA, which suggests an ability to disproportionate thiosulfate to sulfate and hydrogen sulfide (Finster et al., 1998). MAGs did not encode homologs of other terminal oxidases. Homologs of the RNA complex and a F-type ATPase were encoded, providing a mechanism to generate a membrane potential and ion gradient and form ATP, respectively. Homologs of RNF and F-type ATPase pathways were present. RNF links the ferredoxin (Fd) and NADH pools with the ion motive force. When the concentration of Fd is greater than NAD^+^, electron flow is to NAD^+^ and this is coupled to ion translocation out of the cell, conserving energy (Westphal et al., 2018). When NADH is greater than ferredoxin, Rnf works in reverse. The presence of the complete Wood-Ljungdahl pathway and several [NiFe]-hydrogenase homologs suggests that OTU-6 is a hydrogenotrophic autotroph and is likely an acetogen, based on the lack of methanogenesis pathways and other terminal oxidases.

#

# OTU 7: (B) *Desulfohalobiaceae* (f)

Estimated genome size (0 cm depth) = 3.11 Mbp; completeness = 93.3%; contamination = 0.6%

Relative abundance within community = 4.9% at 0 cm, 5.6% at 5 cm, and 2.4% at 30 cm

MAGs corresponding to OTU 7 were identified in all depth intervals of the sediment column in the NA of GSL. OTU 7 is affiliated with the bacterial family *Desulfohalobiaceae*, and encodes a nearly complete Wood-Ljungdahl pathway, missing only those genes encoding formate-tetrahydrofolate ligase and methylenetetrahydrofolate dehydrogenase. The MAG also encodes a nearly complete TCA cycle and a nearly complete pentose phosphate pathway, indicating it is possibly a facultative autotroph, although it lacks a complete glycolytic pathway. Homologs of proteins involved in respiration of sulfate, including sulfate adenylyltransferase (Sat), adenylylsulfate reductase (Aps), and dissimilatory sulfite reductase (DsrAB) were encoded in the MAGs. Additionally, MAGs encode a group 1c [NiFe] hydrogenase and genes corresponding to arsenate reduction (ArrA). MAGs encode homologs of an F-type ATPase, a cytochrome *bd* ubiquinol oxidase (CydAB), a succinate dehydrogenase (Complex II), and a *cbb3*-type cytochrome *c* oxidase (CcoNOP), indicating it is possibly O_2_ tolerant (Pitcher & Watmough, 2004) but unlikely to be capable of respiring O_2_. OTU 7 is likely an O_2_ tolerant anaerobe and facultative autotroph, which is common among members of this bacterial family (Kuever, 2014).

# OTU 8: (A) *Haloamina* (g)

Estimated genome size (0 cm depth) = 2.85 Mbp; completeness = 94.5%; contamination = 1.2%

Relative abundance within community = 2.3% at 0 cm. and 2.4% at 5 cm

MAGs corresponding to OTU 8 were identified in the 0 and 5 cm depth intervals of the sediment column in the NA of GSL. Homologs of autotrophic pathways were not identified. MAGs encode the three-carbon portion of the glycolytic pathway, a nearly complete gluconeogenic pathway, and a nearly complete TCA cycle. Genes encoding for proteins involved in sulfur oxidation (Sdo) were also encoded. In addition, *Haloamina* MAGs encode homologs of the *caa3*-type cytochrome *c* oxidase (CoxAB), succinate dehydrogenase (Complex II), and a V/A-type ATPase. Homologs of other terminal oxidases were not identified. MAGs encode for formate dehydrogenase (FDH), suggesting an ability to oxidize formate. These data suggest OTU 8 is an aerobic heterotroph, consistent with the metabolism of other members of the archaeal order *Halobacteriales* (Oren, 2006).

# OTU 9: (A) *Halovenus* (g)

Estimated genome size (5 cm depth) = 3.0 Mbp; completeness = 86.3%; contamination = 1.7%

Relative abundance within community = 1.8% 0 cm; 1.9% at 5 cm

MAGs belonging to the OTU 9 were identified in the 0 and 5 cm depth intervals in the sediment column of the NA of the GSL. MAGs did not encode autotrophic pathways but did encode a nearly complete glycolytic pathway and TCA cycle. MAGs encoded homologs of *caa3*-type cytochrome c oxidase (CoxAB), suggesting an ability to respire O_2_. MAGs also encoded homologs of nitrite reductase (NirSK) suggesting an ability to use nitrite as an electron acceptor. Homologs of other terminal oxidases were not identified. MAGs encoded a homolog of Sdo that is putatively involved in sulfur oxidation or detoxification (see OTU 1 description). As such, OTU 9 was conservatively designated as a facultatively anaerobic heterotroph with the possibility of a mixotrophic energy metabolism (oxidation of sulfur compounds to supplement organic carbon as an electron donor).

#

# OTU 10: (A) QS-5-70-15 (g)

Estimated genome size (0 cm depth) = 3.61 Mbp; completeness = 77.9%; contamination = 0.9%;

Relative abundance within community= 4.2% at 0 cm, 3.6% at 5 cm. and 3.3% at 30 cm.

MAGs belonging to the OTU 10 were identified at all depths in the sediment column of the NA of GSL. OTU 10 is most closely affiliated with the genus QS-5-70-15 of the archaeal order *Halobacteriales*. MAGs encode nearly complete glycolytic and gluconeogenic pathways, a complete TCA cycle, and a complete pentose phosphate pathway. MAGs did not encode for autotrophic pathways. Additionally, genes encoding proteins involved in sulfur oxidation (Sdo) were identified. MAGs encoded homologs of the *caa3*-type cytochrome *c* oxidase (CoxAB), cytochrome *bd* ubiquinol oxidase (CydAB), succinate dehydrogenase (Complex II), and V/A-type ATPases. Additionally, MAGs encode homologs of carbon monoxide dehydrogenase (CODH), indicating an ability to oxidize carbon monoxide. As such, OTU 10 is designated as an aerobic heterotroph, consistent with other cultivated and characterized members of the *Halobacteriales* (Oren, 2006).

# OTU 11: (A) *Haloarculaceae* (f)

Estimated genome size (5 cm depth) = 2.89 Mbp; completeness = 95.5%; contamination = 2.1%

Relative abundance within community = 2.1% at 0 cm; 1.9% at 5 cm

MAGs belonging to the OTU 11 were identified in the 0 and 5 cm depth intervals in the sediment column of the NA of the GSL. MAGs did not encode autotrophic pathways but did encode complete glycolytic and tricarboxylic acid (TCA) cycles. Homologs of the *caa3*-type cytochrome *c* oxidase (CoxAB) and cytochrome *bd* ubiquinol oxidase (CydAB) implies the potential for O_2_ respiration in the OTU 11. MAGs encoded a homolog of Sdo that is putatively involved in sulfur oxidation or detoxification (see OTU 1 description). As such, OTU 11 was conservatively designated as an aerobic heterotroph with the possibility of a mixotrophic energy metabolism (oxidation of sulfur compounds to supplement organic carbon as an electron donor).

#

# OTU 12: (B) *Salinibacter* (g)

Estimated genome size (0 cm depth) = 3.24 Mbp; completeness = 92.7%; contamination = 1.2%

Relative abundance within community = 5.4% at 0 cm; 4.0% at 5 cm.

MAGs affiliated with OTU 12 were identified in the 0 and 5 cm depth intervals in the sediment column of the NA of the GSL. MAGs did not encode homologs for autotrophic pathways but did encode for complete glycolytic and the pentose phosphate pathways. MAGs also encoded homologs of cytochrome *c* oxidase, both *caa3*- and *cbb3*-types (CoxAB and CcoNOP, respectively), suggesting an ability to respire O_2_. Homologs of other terminal oxidases were not identified. Notably, OTU 12 also harbors genes involved in the oxidation or detoxification of sulfur compounds (Sdo, Sor; see description of OTU 1). Overall, these findings suggest that OTU 12 represents an aerobic heterotroph, with the possibility of a mixotrophic energy metabolism (oxidation of sulfur compounds to supplement organic carbon as an electron donor).

#

# OTU 13: (A) *Natronomonas* (g)

Estimated genome size (0 cm depth) = 2.50 Mbp; completeness = 93.5%; contamination = 3.4% Relative abundance within community = at 3.0% 0 cm; 3.0% at 5 cm

MAGs affiliated with OTU 13 were identified in the 0 and 5 cm depth intervals in the sediment column of the NA of the GSL. Though the MAGs do not encode autotrophic pathways, partially complete TCA cycles and the glycolysis 3-carbon module are present. The OTU encodes for alanine-glycoxylate transaminase (Agxt2) and aspartate aminotransferase homologs in addition to a partially complete leucine degradation pathway that feeds into the TCA cycle. As was studied in other *Natronomonas* genomes, the presence of these genes indicates the OTU likely oxidizes amino acids as a primary carbon source (Falb et al., 2005). MAGs encode the *caa3*-type cytochrome *c* oxidase (CoxAB) and cytochrome *bd* ubiquinol oxidase (CydAB), the latter of which is often found in aerobic bacteria growing in areas of limited O_2_ concentration (Borisov et al., 2011). MAGs also encode a NADP^+^-dependent and copper-containing nitrite reductase, suggesting an ability to respire nitrite. Homologs of other terminal oxidases were not identified. Notably, MAGs also encode homologs of proteins in the oxidation or detoxification of sulfur compounds (Sdo, Sor; see description of OTU 1). Finally, there are genes present for apocarotenoid-15,15'-oxygenase, a protein involved in retinol synthesis and green-light absorbing proteorhodopsin (Kloer et al., 2005), possibly indicating an alternative way of making ATP. Collectively, these observations indicate OTU 13 is a facultatively anaerobic heterotroph, with the possibility of a mixotrophic energy metabolism (oxidation of sulfur compounds to supplement organic carbon as an electron donor). This is consistent with other characterized *Natronomonas* which tend to be aerobic heterotrophs (Falb et al., 2005).

OTU 14: (A) *Haloarculaceae* (f)

Estimated genome size (0 cm depth) = 3.1 Mbp; completeness = 91.4%; contamination = 4.1%

Relative abundance within community = 1.9% at 0 cm

A MAG affiliated with OTU 14 was identified in the 0 cm depth interval of the sediment column from the NA of GSL. OTU 14 is likely an aerobic heterotroph capable of oxidizing nitrite. OTU 14 encodes nearly complete glycolysis (missing: hexokinase, 6-phosphofructokinase), gluconeogenesis (missing: triosephosphate isomerase) and TCA (missing: malate dehydrogenase) pathways/cycles. Additionally, the presence of a homolog of the *caa3*-type cytochrome *c* oxidase (CoxAB) suggests this organism is an aerobe. This organism also encodes genes for aerobic nitrite oxidation (NxrAB and NirK) and carbon monoxide (CODH) oxidation. Collectively, this evidence suggests that this organism is an aerobic heterotroph that might supplement its energy metabolism by oxidizing nitrite or carbon monooxide.

# OTU 15: (B) *Salinivenus* (g)

Estimated genome size (0 cm depth): 3.63 Mbp; completeness = 89.2%; contamination = 0.6% Relative abundance within community = 10.4% at 0 cm; 6.1% at 5 cm.

MAGs associated with OTU 15 were identified in the 0 and 5 cm depth intervals in the sediment column of the NA of the GSL. MAGs do not encode autotrophic pathways but do encode nearly complete glycolytic and gluconeogenic pathways, a complete TCA cycle, and a pentose phosphate pathway. The presence of a homolog of F-type ATPase was detected. MAGs also encode homologs of the *caa3*-type cytochrome *c* oxidase (CoxAB), indicating an ability to respire O_2_. The MAGs also encode a homolog of nitrous oxide reductase (NosZ) which catalyzes the reduction of nitrous oxide (N_2_O) to dinitrogen gas (N_2_) during the denitrification process. OTU 16 is designated as a facultatively anaerobic heterotroph.

# OTU 16: (A) *Haloarculaceae* (f)

Estimated genome size (0 cm depth) = 2.50 Mbp; completeness = 85.3%; contamination = 3.4%; Relative abundance within community = 1.6% at 0 cm, 2% at 5 cm, and 2.9% at 30 cm

MAGs associated with OTU 16 were identified at all depths in the sediment column of the NA of the GSL. MAGs do not encode homologs of autotrophic pathways but do encode a complete TCA cycle and partially complete glycolysis pathway. The MAGs also encode a V/A-type ATPase and homologs of the *caa3*-type cytochrome *c* oxidase (CoxAB), indicating an ability to respire O_2_. The MAGs also encode homologs of nitrite reductase (NirKS) indicating that nitrite can also be respired. OTU 16 is designated as a facultatively anaerobic heterotroph.

# OTU: 17 (A) *Halapricum* (g)

Estimated genome size (0 cm. depth) = 2.65 Mbp; completeness = 83.2%; contamination = 2.0%

Relative abundance within community= 6.2% at 0 cm, 5.7% at 5 cm, and 3.1% at 30 cm.

MAGs associated with OTU 17 were identified at all depths in the sediment column of the NA of GSL. MAGs do not encode homologs of autotrophic pathways but do encode a mostly complete TCA cycle and pentose phosphate pathway, as well as several proteins involved in glycolysis. The MAG encodes homologs of the *caa3*-type cytochrome *c* oxidase (CoxAB), cytochrome *bd* ubiquinol oxidase (CydAB), and NADH quinone oxidoreductase, suggesting an ability to respire O_2_. Homologs of other terminal oxidases were also not identified. OTU 17 encodes homologs enabling lactate fermentation, which is corroborated by the presence of homologs of a group 3 [NiFe] hydrogenase suggesting the ability to produce hydrogen as an end-product of fermentation. Consistent with what was reported by (Sorokin et al., 2021) the MAGs detected from GSL encode for proteins associated with dissimilatory sulfur oxidation (Sdo). OTU 17 is thus classified as an aerobic heterotroph with the ability to ferment, consistent with several other members of this group of organisms (Sorokin et al., 2021). It may also be able to supplement its aerobic energy metabolism through oxidation of sulfide.

# OTU 18: (A) PWKY01 (f)

Estimated genome size 30 cm depth = 1.74 Mbp; completeness = 90.1%; contamination = 1.2% Relative abundance within community = 1.1% at 5 cm; 6.6% at 30 cm

MAGS corresponding to OTU 18 were identified both at the 5 cm and 30 cm depth of the sediment column in the NA of GSL. This OTU is affiliated with the PWKY01 family within the archaeal *Thermoplasmatota* phylum. The MAGs lack homologs of both type *caa3*- and *cbb3*-type cytochrome *c* oxidase (CoxAB, CcoNOP, respectively) and homologs of cytochrome *bd* complex (CydABX), indicating that they are obligately anaerobic. The MAGs encode a V/A-type ATPase. MAGs encode a nearly complete glycolytic pathway and a nearly complete archaeal PPP pathway but lack a complete TCA cycle. GSL MAGs encode genes for HdrABC/MvhAGD, a cytoplasmic heterodisulfide reductase-[NiFe]-hydrogenase complex that links Fd reduction and heterodisulfide reduction with H_2_ oxidation. GSL MAGs also encode homologs of a group [NiFe]-hydrogenases. As such, we propose that OTU 18 is an anaerobic heterotroph and is likely fermentative.

# OTU 19: (B) *Ca. Bipolaricaulia* (c)

# Estimated genome size (30 cm depth) = 1.7 Mbp; completeness = 82.2%; contamination = 0.0%  Relative abundance within community = 1.7% at 5cm; 8.2% at 30cm.

# ​​MAGs corresponding to OTU 19 were recovered from two different depths (5 and 30 cm) in the sediment column from the NA of GSL. MAGs encoded a complete reductive acetyl-CoA pathway (Wood-Ljungdahl pathway) suggesting it is capable of autotrophy. Homologs of RNF and F-type ATPase pathways were present. RNF links the ferredoxin (Fd) and NADH pools with the ion motive force. When the concentration of Fd is greater than NAD^+^, electron flow is to NAD^+^ and this is coupled to ion translocation out of the cell, conserving energy (Westphal et al., 2018). When NADH is greater than ferredoxin, Rnf works in reverse. Homologs of several [NiFe]-hydrogenases (Groups 1a, 4a, and 4g, based on the HydDB classification tool (Sondergaard et al., 2016)). These are predicted to allow for the generation of reduced Fd and NADPH. MAGs also encoded homologs of PhsA, which disproportionates thiosulfate to sulfate and hydrogen sulfide (Finster et al., 1998). Homologs of other terminal oxidases were not identified. OTU 19 is classified as an anaerobic hydrogenotrophic autotroph, more specifically an acetogen given the absence of evidence of methanogenesis pathways (e.g., Mcr) and other terminal oxidases.

# OTU 20: (B) UBA12077 (f)

Estimated genome size (5 cm depth) = 2.82 Mb; completeness = 89.2%; contamination = 2.4%

Relative abundance within community = 1.4% at 0 cm, 1.6% at 5 cm, and 3.4% at 30 cm

MAGs affiliated with OTU 20 were detected at multiple sediment depths (0, 5, and 30 cm) within the sediment column from the NA of the GSL. OTU 20 could only be classified to the UBA12077 family of the *Bacteroidales* order. The MAGs encoded a mostly complete glycolytic, gluconeogenic, and tricarboxylic acid (TCA) pathway/cycle. MAGs did not encode complete carbon fixation pathways, although several homologs of proteins involved in the Wood-Ljungdahl (WL) pathway (MetF, MetV, RnfC2, FoID, AckA, and FchA) were identified. These observations suggest a heterotrophic metabolism for OTU 20. Homologs of terminal oxidases, such as cytochrome *c* oxidase (CoxAB), were not identified in the MAGs. However, the presence of a homolog of lactate dehydrogenase homolog (Ldh) in the MAGs indicates their capacity for carrying out fermentation. The presence of several homologs for both F-type and V/A-type ATPase subunits was detected. Homologs of several [FeFe] hydrogenases were also identified, indicating the presence of enzymes capable of catalyzing the production or consumption of H_2_, perhaps consistent with the ability to ferment lactate. Collectively, OTU 20 is depicted as likely being an anaerobic fermenter.

#

# OTU 21: (A) *Haloarculaceae* (f)

Estimated genome size (0 cm depth) = 2.1 Mbp; completeness = 74.8%; contamination = 0.5%

Relative abundance within community = 2.3% at 0 cm; 1.9% at 5 cm

MAGs affiliated with the OTU-21 were recovered from the 0 and 5 cm depths of the sediment column from the NA of GSL. MAGs did not encode homologs of autotrophic pathways but did encode for partial glycolytic, TCA, and gluconeogenesis pathways/cycles. The lack of complete pathways is likely a reflection of the relative incompleteness of the MAG (estimated completeness = 74.8%). MAGs encoded homologs of the *caa3*-type cytochrome *c* oxidase (CoxAB), suggesting an ability to respire _O2._ Similarly, homologs of nitrite reductase (NirKS) were also identified, suggesting an ability to utilize nitrite as an electron acceptor. MAGs did not encode homologs of other terminal oxidases. The presence of homologs of formate dehydrogenase proteins (FdoG, FdwB, FdoH, FdhAB) suggests an ability for cells to oxidize formate. These observations collectively suggest that OTU 21 is a facultatively anaerobic heterotroph.

#

# OTU 22: (A) *Halobacteriales* (o)

Estimated genome size (0 cm depth) = 1.9 Mbp; completeness = 67.4%; contamination = 1.2%

Relative abundance within community = 1.5% at 0 cm; 1.1% at 5 cm

MAGs attributed to OTU 22, which were found in 0 and 5 cm sediment depths of the NA of GSL, lack several homologs associated with central carbon metabolism pathways such as glycolytic and gluconeogenic pathways, except for the presence of a complete pyruvate oxidation pathway. In addition, the MAG did not encode carbon fixation pathways. MAGs encoded homologs of the *caa3*-type cytochrome *c* oxidase (CoxAB), suggesting an ability to respire O_2_. Homologs of other terminal oxidases were not identified. MAGs encoded homologs of proteins involved in the oxidation or detoxification of sulfur compounds (Sdo; see description of OTU 1). MAGs displayed an absence of homologs related to nitrogen metabolism, and no homologs for [NiFe] and [FeFe] hydrogenases were detected. Collectively, these observations indicate OTU 22 is an aerobic heterotroph, with the possibility of mixotrophic energy metabolism (oxidation of sulfur compounds to supplement organic carbon as an electron donor).

# OTU 23: (A) *Halovenus* sp004015825 (s)

Estimated genome size (0 cm depth) = 1.68 Mbp; completeness = 64.9%; contamination = 0.6%

Relative abundance within community = 1.3% at 0 cm; 1.1% at 5 cm

MAGs corresponding to OTU 23 were recovered from the 0 and 5 cm depths of the sediment column from the NA of GSL. MAGs did not encode homologs of carbon fixation pathways but did encode the complete three carbon compound core module of glycolysis. MAGs encoded homologs of the *caa3*-type cytochrome *c* oxidase (CoxAB) and a V/A-type ATPase, suggesting ATP production via respiration of O_2_. Homologs of other terminal oxidases were not identified; however, the MAG encodes proteins allowing for sulfur oxidation (Sdo) and DMSO oxidation. OTU 23 is 65% complete, so important aspects of its metabolism may be missing from this analysis. OTU 23 is conservatively classified as an aerobic heterotroph, with the possibility of a mixotrophic energy metabolism (oxidation of sulfur compounds to supplement organic carbon as an electron donor). This classification is consistent with other characterized isolates of *Halovenus*, which are obligate aerobes that can metabolize a variety of simple and complex carbon compounds (Makhdoumi-Kakhki et al., 2012).

#

# OTU 24: (A) *Haloarcula* (g)

Estimated genome size (5 cm depth) = 2.30 Mbp; completeness = 43.2%; contamination = 0.4% Relative abundance within community = 2.7% at 0 cm; 1.6% at 5 cm

MAGs corresponding to OTU 24 were recovered from sediment samples at depths of 0 and 5 cm in the NA of GSL. The MAGs encode most of the homologs for glycolysis, gluconeogenesis, and pyruvate oxidation pathways but did not encode homologs of autotrophic pathways. Homologs of the *caa3*-type cytochrome *c* oxidase (CoxAB) and cytochrome *bd* quinone oxidase (CydAB) were encoded, signifying the ability to perform aerobic respiration. A homolog of nitrite reductease (NirK) was detected, indicating an ability to reduce nitrite (NO_2_^-^) to nitric oxide (NO) during microbial nitrogen metabolism. No homologs of F-type ATPases or V/A-type ATPases were identified, which may be attributed to the incompleteness level of the MAG (43.2%). Based on the available data and classification, OTU 24 was classified as an aerobic heterotroph.

#

# OTU 25: (A) DHVEG-1 (f)

Estimated genome size (30 cm depth) = 2.92 Mbp; completeness = 87.9%; contamination = 0.0%

Relative abundance within community = 0% at 0 cm, 0% at 5 cm, and 9.1% at 30 cm.

MAGS corresponding to OTU 25 were identified only at the 30 cm depth of the sediment column in the NA of GSL. The MAGs lack genes for both type *caa3*- and *cbb3*-type cytochrome *c* oxidase (CoxAB, CcoNOP, respectively), and the cytochrome *bd* complex (CydABX), indicating that they are obligately anaerobic. The MAGs encode a V/A-type ATPase. MAGs encode nearly complete glycolytic and archaeal PPP pathways, though they lack a complete TCA cycle. MAGs also encode for several fermentation pathways, indicating that this GSL DHVEG-1 MAG is capable of heterotrophic metabolism. However, this MAG also encodes the reverse TCA cycle (rTCA), with homologs encoding proteins indicative of this pathway (ATP citrate lyase (AclA), ATP citrate synthetase (CcsA) and citryl-CoA synthetase (CcsB) all present. The designation of anaerobic facultative autotroph is consistent with previous research into the *Thermoprofundales* order, along with MBG-D genomes (Zhou et al., 2019), however, the mechanism of autotrophy is not the same. Other DHVEG-1/*Thermoprofundales* genomes have been found to encode the Wood-Ljungdahl pathway rather than rTCA cycle (Zhou et al., 2019). While these MAGs do encode formate tetrahydrofolate ligase (Fhs), they do not encode a sufficient number of other genes necessary for the WL pathway. As such, it is likely that OTU 25 is a facultative anaerobic autotroph.

# OTU 26: (A) PWHR01(g)

Estimated genome size (30 cm depth) = 2.18 Mbp; completeness = 97.6%; contamination = 1.6% Relative abundance within community = 0% at 0 cm, 0% at 5 cm, and 3.8% at 30 cm.

MAGS corresponding to OTU 26 were identified only in the 30 cm depth of the sediment column in the NA of GSL. PWHR01 designation is a placeholder name for a proposed genus within the PWKY01 order within the Archaea. The MAGs lack genes for both type *caa3*- and *cbb3*-type cytochrome *c* oxidase (CoxAB, CcoNOP, respectively), and the cytochrome *bd* complex (CydABX), indicating that they are obligately anaerobic. MAGs do not encode homologs of alternative terminal oxidases. The MAGs encode a V/A-type ATPase. MAGs encode almost complete glycolytic and a mostly complete archaeal PPP pathway, though they lack a complete TCA cycle, along with several fermentation pathways, indicating that this GSL DHVEG-1 MAG is capable of heterotrophic metabolism. As such, we propose that OTU 26 is an anaerobic heterotroph and is likely fermentative.

# OTU 27: (A) DHVEG-1 (f)

Estimated genome size (30 cm depth) = 1.54 Mbp; completeness = 85.7%; contamination = 1.6%

Relative abundance within community = 0% at 0 cm, 0% at 5 cm, and 3.4% at 30 cm.

A MAG corresponding to OTU 27 was identified only at the 30 cm depth of the sediment column in the NA of GSL. The MAG lacks genes encoding both type *caa3*- and *cbb3*-type cytochrome *c* oxidase (CoxAB, CcoNOP, respectively), and the cytochrome *bd* complex (CydABX), indicating that they are obligately anaerobic. The MAG does not encode homologs of alternative terminal oxidases. MAGs encode complete glycolytic and a mostly complete archaeal PPP pathway, though they lack a complete TCA cycle. The MAGs encode a V/A-type ATPase. No autotrophic pathways are encoded by the MAG, indicating that it likely corresponds to an anaerobic hetertroph. While these MAGs do encode formate tetrahydrofolate ligase (Fhs), they do not encode a sufficient number of other genes necessary for the WL pathway. Interestingly, despite its close relatedness to OTU 25, the MAGs for OTU 27 do not encode any of the necessary genes for the rTCA cycle, providing potential insight into why two similar MAGs (populations) coexist rather than outcompeting one another. OTU 27 has been designated as an anaerobic heterotroph.

# OTU 28: (B) *Bacteroides acidifaciens* (s)

Estimated genome size (30 cm depth) = 4.72 Mbp; completeness = 98.9%; contamination = 0.4%

Relative abundance within community = 26.9% at 30 cm

A MAG affiliated with OTU 28 was identified in the 30 cm depth interval of the sediment column from the NA of GSL. The MAG did not encode homologs of autotrophic pathways but did encode complete glycolytic and pentose phosphate pathways and a mostly complete TCA cycle (missing succinyl-CoA synthetase alpha subunit). Due to the presence of an encoded cytochrome *bd* oxidase (CydAB) and absence of cytochrome *c* oxidases (CoxAB, CcoNOP), the MAG was classified tentatively as aerotolerant. The lack of homologs of alternative terminal oxidases, coupled with the presumed lack of O_2_ at the 30 cm depth in the sediment column indicates that this MAG may operate a fermentative metabolism. Furthermore, the presence of homologs of phosphate acetyl-transferase (Pta) and acetate kinase (Ack) in the MAG suggests that this OTU is capable of consuming acetate in the absence of more favorable fermentable carbon sources (e.g. glucose, (Enjalbert et al., 2017)). Two homologs of [FeFe]-hydrogenases were identified (Group A and B), which likely couple NADH or Fd oxidation with H_2_ production to allow fermentation to continue. For this reason, OTU 28 is classified as a fermentative aerotolerant heterotroph.

# OTU 29: (A) *Haloplanus* (g)

Estimated genome size (0 cm depth) = 1.26 Mbp; completeness = 41.0%; contamination = 6.5%

Relative abundance within community = 1.3% at 0 cm

A MAG affiliated with OTU 29 was identified in 0 cm depth interval of the sediment column from the NA of GSL. The MAG encodes glycolytic and pentose phosphate pathways and encodes the TCA cycle. The MAG also encodes mannan endo-1,4-beta-mannosidase, which can break down endohemicellulases. The GSL MAG also encodes homologs of molybdenum-dependent carbon monoxide dehydrogenase (CODH), potentially enabling CO oxidation. The MAG does not encode autotrophic pathways. The MAG encodes homologs of the *caa3*-type cytochrome *c* oxidase and, along with homologs of cytochrome *bd* ubiquinol oxidase, likely indicates this MAG corresponds to an aerobe. In the literature, *Haloplanus* isolates are aerobes, with a few species able to grow anaerobically using nitrate or DMSO (Bardavid et al., 2007; Han & Cui, 2014a; Qiu et al., 2013). Importantly, this MAG is only 41% complete, so other aspects of its metabolism may be missing from this analysis. As such, this OTU is conservatively designated as an aerobic heterotroph.

# OTU 30: (B) *Paceibacterales* (o)

# Estimated genome size (5 cm depth) = 0.64 Mbp; completeness = 66.9%; contamination = 2.2%

# Relative abundance within community = 1.4% at 5 cm.

# A MAG affiliated with OTU 30 was identified in the 5 cm depth interval of the sediment column from the NA of GSL. OTU 30 is likely a heterotrophic symbiont. It lacks all genes for carbon metabolism except gluconeogenesis and glycolysis, while also encoding genes for the synthesis of ribonucleic acids, and an F-type ATPase. The description of this MAG is consistent with published characterizations of other *Paceibacterales* MAGs that report them to have a small genome (~0.60 Mb) with few annotated functions (Chaudhari et al., 2021). Published *Paceibacterales* MAGs often lack most central energy metabolism and biosynthetic pathways for most amino acids and vitamins, while encoding for F-Type ATPases (Tian et al., 2020). Due to their small genomes and lack of apparent metabolic functions, along with the observation of close association and potential cross-feeding with other microorganisms (specifically methanogens), has led to the general characterization of the described members of *Paceibacterales* as obligate symbionts (Kuroda, 2022).The OTU is thus classified as a heterotroph, although it is unclear if it is an aerobe or an anaerobe.

# OTU 31: (A) *Halofilum* (g)

Estimated genome size (5 cm depth) = 1.23 Mbp; completeness = 47.8%; contamination =3.1%

Relative abundance within community = 1.0% at 5 cm

A MAG associated with OTU 31 was identified at a 5 cm sediment depth within the NA of the GSL. The MAG did not encode a complete glycolytic, gluconeogenic, or tricarboxylic acid (TCA) cycle. Moreover, complete carbon fixation pathways were absent. The majority of the non-oxidative pentose phosphate pathway homologs was present within the MAG. The presence of homologs of cytochrome *c* oxidase (CoxAB) and cytochrome *bd* quinone oxidase (CydAB) homologs was observed, suggesting the potential capability of respiration using O_2_ as an electron acceptor. An F-type ATPase homolog was detected. OTU 31 is conservatively designated as an aerobic heterotroph, although the relatively low completeness level of the genome (47.8%) should be kept in mind.

# OTU 32: (B) *Brocadiae* (c)

Estimated genome size (5 cm depth) = 1.10; Mbp; completeness = 42.3%; contamination = 0.0%

Relative abundance within community = 1.0% at 5 cm

A MAG affiliated with OTU 32 was identified in the 5 cm depth interval of the sediment column from the NA of GSL. The MAG was highly incomplete (42.3%) and, unsurprisingly encodes no complete pathways for central carbon metabolism (glycolysis, TCA cycle). However, it encodes homologs of key enzymes in the Wood-Ljungdahl Pathway (CdhE, CooS), indicating a capacity to fix CO_2_. This MAG also encodes two homologs of [NiFe]-hydrogenase (group 1 and group 3abd, based on the HydDB classification tool). The lack of homologs of cytochrome *c* or cytochrome *bd* oxidase genes suggests that this MAG corresponds to an anaerobe. The MAG does not encode homologs for alternative terminal oxidases. For these reasons, this OTU is classified as an anaerobic autotroph, possibly an acetogen given the lack of alternative terminal oxidases and homologs of methanogenesis proteins (e.g., Mcr).

#

# OTU 33: (A) *Haloferacaceae* (f)

Estimated genome size (5 cm depth) = 2.32 Mpb; completeness = 47.8%; contamination = 3.1%

Relative abundance within community = 1.0% at 5 cm

At a depth of 5 cm in the sediment of the NA of the GSL, a MAG corresponding to OTU 33 was identified that harbors a mostly complete glycolytic pathway, gluconeogenesis pathway, and partial TCA cycle. The MAG does not encode for autotrophic pathways. The MAG encodes homologs of the *caa3*-type cytochrome *c* oxidase (CoxAB) and cytochrome *bd* oxidase (CydAB), suggesting the potential for aerobic respiration. The cytochrome oxidase *bd* complex is known for its low affinity for O_2_ and its ability to function in low O_2_concentrations (Korshunov et al., 2016). The MAG does not encode for other terminal oxidases but encodes a homolog of V/A-type ATPase. Homologs of [NiFe]- and [FeFe]-hydrogenases were not detected. OTU 33 is therefore classified as an aerobic heterotroph.

#

# OTU 34: (A) *Natronomonas* (g)

Estimated genome size (5 cm depth): 1.53 Mbp; completeness = 59.6%; contamination = 3.2%; Relative abundance within community = 1.0% at 5 cm

At a depth of 5 cm in the sediment of the NA of the GSL, a MAG corresponding to OTU 34 was identified. The MAG does not encode autotrophic pathways but does encode incomplete glycolysis and gluconeogenesis pathways and the TCA cycle. Based on previous descriptions of *Natronomonas*, these cells oxidize amino acids as a primary carbon source (Falb et al., 2005). Consistent with this, the GSL MAG encodes a homolog of alanine-glyoxylate transaminase (Agxt2), a protein often found in amino acid oxidation, as well as a nearly complete leucine degradation pathway that feeds into the TCA cycle. MAGs encode proteins for sulfur oxidation (Sdo and Sor), suggesting the possibility that sulfur can contribute to the energy metabolism of the cell. MAGs encode homologs of the *caa3*-type cytochrome *c* oxidase (CoxAB), a V-type ATPase, but no other homologs of terminal oxidases, suggesting the potential for aerobic respiration. Interestingly, the MAG contains genes for opsin-activated proteins, suggesting a role in environmental sensing. The OTU is therefore classified as an aerobic heterotroph.

# OTU 35: (A) *Nanosalinaceae* (f)

Estimated genome size (5 cm depth) = 0.54 Mbp; completeness = 40.1%; contamination = 7.2% Relative abundance within community = 0.9% at 5 cm.

A MAG corresponding to OTU 35 was identified in the 5 cm depth interval in the NA sediment column from GSL. This MAG is mostly incomplete and exhibits a high level of contamination. The family *Nanosalinaceae* is suggested, however, to be a symbiont of other halophilic archaea, especially *Halobacteria* (Zhao et al., 2022). This MAG encodes homologs of several genes in the glycolytic and gluconeogenesis pathways and encodes a V/A-type ATPase. The MAG lacks homologs of autotrophic pathways. Additionally, no terminal oxidases were identified, although this finding could be a result of the incompleteness of this MAG. Together, this suggests that OTU 35 corresponds to a heterotroph and it is likely in a symbiotic relationship with a yet to be identified halophilic archaeon.

# OTU 36: (A) Halobacteriales SW-7-71-33 (f)

Estimated genome size (5 cm depth) = 1.39 Mbp; completeness = 41.8%; contamination = 3.3%; Relative abundance within community= 1.0% at 5 cm.

A MAG corresponding to OTU 36 was identified in the 5 cm depth interval in the NA sediment column from GSL. This MAG, a member of *Halobacteriales* family SW-7-71-33, is mostly incomplete and has a large amount of contamination. As a member of order *Halobacteriales*, it is likely to be an aerobic heterotroph (Oren, 2006). Consistent with this, the MAG did not encode homologs of autotrophic pathways but did encode partial glycolytic and gluconeogenesis pathways, as well as a partial TCA cycle. Additionally, this MAG encodes a group 1a [NiFe] hydrogenase. The MAG encoded homologs of the *caa3*-type cytochrome *c* oxidase, NADH:quinone oxidoreductase (Complex I), and a V/A-type ATPase. The MAG did not encode homologs of other terminal oxidases. OTU 36 is classified as an aerobic heterotroph.

**References**

Bardavid, R. E., Mana, L., & Oren, A. (2007). *Haloplanus natans* gen. nov., sp. nov., an extremely halophilic, gas-vacuolate archaeon isolated from Dead Sea-Red Sea water mixtures in experimental outdoor ponds. *Int J Syst Evol Microbiol*, *57*(Pt 4), 780-783. <https://doi.org/10.1099/ijs.0.64648-0>

Bardiya, N., & Bae, J. H. (2011). Dissimilatory perchlorate reduction: a review. *Microbiol Res*, *166*(4), 237-254. <https://doi.org/10.1016/j.micres.2010.11.005>

Borisov, V. B., Gennis, R. B., Hemp, J., & Verkhovsky, M. I. (2011). The cytochrome bd respiratory oxygen reductases. *Biochim Biophys Acta*, *1807*(11), 1398-1413. <https://doi.org/10.1016/j.bbabio.2011.06.016>

Burns, D. G., Janssen, P. H., Itoh, T., Kamekura, M., Li, Z., Jensen, G., Rodriguez-Valera, F., Bolhuis, H., & Dyall-Smith, M. L. (2007). *Haloquadratum walsbyi* gen. nov., sp. nov., the square haloarchaeon of Walsby, isolated from saltern crystallizers in Australia and Spain. *Int J Syst Evol Microbiol*, *57*(Pt 2), 387-392. <https://doi.org/10.1099/ijs.0.64690-0>

Chaudhari, N. M., Overholt, W. A., Figueroa-Gonzalez, P. A., Taubert, M., Bornemann, T. L. V., Probst, A. J., Holzer, M., Marz, M., & Kusel, K. (2021). The economical lifestyle of CPR bacteria in groundwater allows little preference for environmental drivers. *Environ Microbiome*, *16*(1), 24. <https://doi.org/10.1186/s40793-021-00395-w>

Enjalbert, B., Millard, P., Dinclaux, M., Portais, J. C., & Letisse, F. (2017). Acetate fluxes in *Escherichia coli* are determined by the thermodynamic control of the Pta-AckA pathway. *Sci Rep*, *7*, 42135. <https://doi.org/10.1038/srep42135>

Falb, M., Pfeiffer, F., Palm, P., Rodewald, K., Hickmann, V., Tittor, J., & Oesterhelt, D. (2005). Living with two extremes: conclusions from the genome sequence of *Natronomonas pharaonis*. *Genome Res*, *15*(10), 1336-1343. <https://doi.org/10.1101/gr.3952905>

Finster, K., Liesack, W., & Thamdrup, B. (1998). Elemental sulfur and thiosulfate disproportionation by *Desulfocapsa sulfoexigens* sp. nov., a new anaerobic bacterium isolated from marine surface sediment. *Appl Environ Microbiol*, *64*(1), 119-125. <https://doi.org/10.1128/AEM.64.1.119-125.1998>

Han, D., & Cui, H. L. (2014a). *Haloplanus litoreus* sp. nov. and *Haloplanus ruber* sp. nov., from a marine solar saltern and an aquaculture farm, respectively. *Antonie Van Leeuwenhoek*, *105*(4), 679-685. <https://doi.org/10.1007/s10482-014-0123-6>

Han, D., & Cui, H. L. (2014b). *Halosimplex pelagicum* sp. nov. and *Halosimplex rubrum* sp. nov., isolated from salted brown alga Laminaria, and emended description of the genus Halosimplex. *Int J Syst Evol Microbiol*, *64*(Pt 1), 169-173. <https://doi.org/10.1099/ijs.0.056887-0>

Kloer, D. P., Ruch, S., Al-Babili, S., Beyer, P., & Schulz, G. E. (2005). The structure of a retinal-forming carotenoid oxygenase. *Science*, *308*(5719), 267-269. <https://doi.org/10.1126/science.1108965>

Korshunov, S., Imlay, K. R., & Imlay, J. A. (2016). The cytochrome bd oxidase of *Escherichia coli* prevents respiratory inhibition by endogenous and exogenous hydrogen sulfide. *Mol Microbiol*, *101*(1), 62-77. <https://doi.org/10.1111/mmi.13372>

Kuever, J. (2014). The Family Desulfohalobiaceae. In E. Rosenberg, E. F. DeLong, S. Lory, E. Stackebrandt, & F. Thompson (Eds.), *The Prokaryotes: Deltaproteobacteria and Epsilonproteobacteria* (pp. 87-95). Springer Berlin Heidelberg. <https://doi.org/10.1007/978-3-642-39044-9_311>

Kuroda, Y. (2022). Remarkable solvent effect of fluorinated alcohols on azo-ene reactions. *Chem Pharm Bull (Tokyo)*, *70*(5), 359-361. <https://doi.org/10.1248/cpb.c22-00076>

Makhdoumi-Kakhki, A., Amoozegar, M. A., & Ventosa, A. (2012). *Halovenus aranensis* gen. nov., sp. nov., an extremely halophilic archaeon from Aran-Bidgol salt lake. *Int J Syst Evol Microbiol*, *62*(Pt 6), 1331-1336. <https://doi.org/10.1099/ijs.0.031419-0>

Oren, A. (2006). The Order Halobacteriales. In M. Dworkin, S. Falkow, E. Rosenberg, K.-H. Schleifer, & E. Stackebrandt (Eds.), *The Prokaryotes: Volume 3: Archaea. Bacteria: Firmicutes, Actinomycetes* (pp. 113-164). Springer New York. <https://doi.org/10.1007/0-387-30743-5_8>

Oren, A., Gurevich, P., Gemmell, R. T., & Teske, A. (1995). *Halobaculum gomorrense* gen. nov., sp. nov., a novel extremely halophilic archaeon from the Dead Sea. *Int J Syst Bacteriol*, *45*(4), 747-754. <https://doi.org/10.1099/00207713-45-4-747>

Pitcher, R. S., & Watmough, N. J. (2004). The bacterial cytochrome *cbb3* oxidases. *Biochimica et Biophysica Acta (BBA) - Bioenergetics*, *1655*, 388-399. <https://doi.org/https://doi.org/10.1016/j.bbabio.2003.09.017>

Qiu, X. X., Zhao, M. L., Han, D., Zhang, W. J., & Cui, H. L. (2013). *Haloplanus salinus* sp. nov., an extremely halophilic archaeon from a Chinese marine solar saltern. *Arch Microbiol*, *195*(12), 799-803. <https://doi.org/10.1007/s00203-013-0929-z>

Sondergaard, D., Pedersen, C. N., & Greening, C. (2016). HydDB: A web tool for hydrogenase classification and analysis. *Sci Rep*, *6*, 34212. <https://doi.org/10.1038/srep34212>

Sorokin, D. Y., Messina, E., Smedile, F., Roman, P., Damste, J. S. S., Ciordia, S., Mena, M. C., Ferrer, M., Golyshin, P. N., Kublanov, I. V., Samarov, N. I., Toshchakov, S. V., La Cono, V., & Yakimov, M. M. (2017). Discovery of anaerobic lithoheterotrophic haloarchaea, ubiquitous in hypersaline habitats. *ISME J*, *11*(5), 1245-1260. <https://doi.org/10.1038/ismej.2016.203>

Sorokin, D. Y., Yakimov, M. M., Messina, E., Merkel, A. Y., Koenen, M., Bale, N. J., & Sinninghe Damsté, J. S. (2021). *Halapricum desulfuricans* sp. nov., carbohydrate-utilizing, sulfur-respiring haloarchaea from hypersaline lakes. *Systematic and Applied Microbiology*, *44*(6), 126249. <https://doi.org/https://doi.org/10.1016/j.syapm.2021.126249>

Tian, Y., Gui, W., Koo, I., Smith, P. B., Allman, E. L., Nichols, R. G., Rimal, B., Cai, J., Liu, Q., & Patterson, A. D. (2020). The microbiome modulating activity of bile acids. *Gut Microbes*, *11*(4), 979-996. <https://doi.org/10.1080/19490976.2020.1732268>

Waino, M., Tindall, B. J., & Ingvorsen, K. (2000). *Halorhabdus utahensis* gen. nov., sp. nov., an aerobic, extremely halophilic member of the Archaea from Great Salt Lake, Utah. *Int J Syst Evol Microbiol*, *50 Pt 1*, 183-190. <https://doi.org/10.1099/00207713-50-1-183>

Wang, H., Liu, S., Liu, X., Li, X., Wen, Q., & Lin, J. (2014). Identification and characterization of an ETHE1-like sulfur dioxygenase in extremely acidophilic *Acidithiobacillus* spp. *Appl Microbiol Biotechnol*, *98*(17), 7511-7522. <https://doi.org/10.1007/s00253-014-5830-4>

Westphal, L., Wiechmann, A., Baker, J., Minton, N. P., & Muller, V. (2018). The Rnf complex is an energy-coupled transhydrogenase essential to reversibly link cellular NADH and ferredoxin pools in the acetogen *Acetobacterium woodii*. *J Bacteriol*, *200*(21). <https://doi.org/10.1128/JB.00357-18>

Wu, W., Pang, X., Lin, J., Liu, X., Wang, R., Lin, J., & Chen, L. (2017). Discovery of a new subgroup of sulfur dioxygenases and characterization of sulfur dioxygenases in the sulfur metabolic network of *Acidithiobacillus caldus*. *PLoS One*, *12*(9), e0183668. <https://doi.org/10.1371/journal.pone.0183668>

Zhang, J., Liu, R., Xi, S., Cai, R., Zhang, X., & Sun, C. (2020). A novel bacterial thiosulfate oxidation pathway provides a new clue about the formation of zero-valent sulfur in deep sea. *ISME J*, *14*(9), 2261-2274. <https://doi.org/10.1038/s41396-020-0684-5>

Zhao, D., Zhang, S., Kumar, S., Zhou, H., Xue, Q., Sun, W., Zhou, J., & Xiang, H. (2022). Comparative genomic insights into the evolution of *Halobacteria*-associated "*Candidatus Nanohaloarchaeota*". *mSystems*, *7*(6), e0066922. <https://doi.org/10.1128/msystems.00669-22>

Zhou, Z., Liu, Y., Lloyd, K. G., Pan, J., Yang, Y., Gu, J. D., & Li, M. (2019). Genomic and transcriptomic insights into the ecology and metabolism of benthic archaeal cosmopolitan, *Thermoprofundales* (MBG-D archaea). *ISME J*, *13*(4), 885-901. <https://doi.org/10.1038/s41396-018-0321-8>
